# Bone spoons for prehistoric babies: Detection of human teeth marks on the Neolithic artefacts from the site Grad-Starčevo (Serbia)

**DOI:** 10.1371/journal.pone.0225713

**Published:** 2019-12-19

**Authors:** Sofija Stefanović, Bojan Petrović, Marko Porčić, Kristina Penezić, Jugoslav Pendić, Vesna Dimitrijević, Ivana Živaljević, Sonja Vuković, Jelena Jovanović, Sanja Kojić, Andrej Starović, Tamara Blagojević

**Affiliations:** 1 BioSense Institute, University of Novi Sad, Novi Sad, Serbia; 2 Laboratory for Bioarchaeology, Department of Archaeology, Faculty of Philosophy, University of Belgrade, Belgrade, Serbia; 3 Dentistry Clinic of Vojvodina, Faculty of Medicine, University of Novi Sad, Novi Sad, Serbia; 4 Faculty of Technical Sciences, University of Novi Sad, Novi Sad, Serbia; 5 National Museum in Belgrade, Belgrade, Serbia; University at Buffalo - The State University of New York, UNITED STATES

## Abstract

Around 8000 years ago, throughout the Neolithic world a new type of artefact appeared, small spoons masterly made from cattle bone, usually interpreted as tools, due to their intensive traces of use. Contrary to those interpretations, the small dimensions of spoons and presence of intensive traces of use led us to the assumption that they were used for feeding babies. In order to test that assumption we compared 2230 marks on three spoons from the Neolithic site of Grad-Starčevo in Serbia (5800−5450 cal BC) with 3151 primary teeth marks produced experimentally. This study has shown that some of the marks on spoons were made by primary teeth, which indicate their usage in feeding babies. The production of a new type of artefact to feed babies is probably related to the appearance of a new type of weaning food, and the abundance of spoons indicates that new baby gruels became an important innovation in prehistoric baby-care.

## Introduction

One of the major turning points in human history was the process of Neolithization, which brought about important changes in the lifestyle of prehistoric people. A series of transformations, such as a more sedentary way of life, plant and animal domestication were followed by the ‘Neolithic Demographic transition’. The Neolithic Demographic Transition refers to the increase of fertility in farming communities in prehistory which made a dramatic impact on the demography of Neolithic populations [1]. The current assumption on the causes which enabled women have more children include several changes in maternal behavior: reduced mobility (increased sedentism), a shift towards consumption of higher calorie cereal food and a reduction of the length of lactation [2]. As a consequence of those changes, the increase in the number of babies resulted in a rapid and unprecedented population growth [3]. Although prehistoric mothers and babies represent the key pillars of demographic success, their role in this process has not been adequately studied, either by archaeologists or physical anthropologists. The reason is rooted in the development of both archeology and anthropology in which children and women were for a long time out of the focus of researchers. With the development of gender archaeology from the 1970s, the studies on women have increased, and from the 1990s a more focused approach appeared in the study of the lives of children in the past [4, 5, 6]. During the last decades, the research on children and childhood has been constantly increasing both in archaeology and bioarchaeology [for a review see 7, 8, 9]. Although a proliferation of studies on the lives of women and children in the past is evident, the investigation of sociobiological phenomena which are relevant for human survival and which include both women and children, such as motherhood, are still very rare. Only recently have prehistoric mothers and babies started to become studied through scientific programs which investigate biological and cultural aspects of prehistoric motherhood, such as ongoing ERC BIRTH project. However, the lack of emphasis on prehistoric motherhood in the history of archaeological research has serious consequences for our current very fragmentary knowledge about biological and cultural mechanisms behind the Neolithic demographic success. This demographic success probably brought about profound changes in prehistoric motherhood, as the increased number of babies demanded new daily life routines not only for prehistoric parents but for the whole community. Thus, by further investigation of prehistoric motherhood, it is possible to gain new insights into the causes of the first significant fertility increase in humans.

### Prehistoric motherhood and novel weaning choices in the Neolithic

Some of the most important changes in prehistoric motherhood in the Neolithic were probably the changes in the duration of breastfeeding and novel weaning food choices aided by the appearance of new agricultural products. There is a correlation between the duration of breastfeeding and fertility, because the production of breast milk is an important energetic investment for the mother which delays ovulation during a period of time, known as the lactational amenorrhea or post-partum amenorrhea [10, 11]. Thus, prolonged breastfeeding with frequent suckling may extend the interval between births and reduce the number of children per female [12, 13, 14]. For this reason, some researchers previously suggested that one of the causes of the fertility increase in the Neolithic was the shortening of the duration of breastfeeding as a consequence of the appearance of domesticated cereals and animal milk, which enabled earlier weaning [15, 16, 17]. But this “weaning foods availability” hypothesis is mainly rejected, because the study of ethnographic evidence showed that although farming populations breastfed for a shorter time than hunter-gatherers, this tendency was not universal and many agricultural populations breastfed as long as some hunter-gatherer populations [18, 19]. From our point of view, there are two important reasons why it is still too early for the rejection of the “weaning foods” hypothesis. First, direct skeletal evidence for the duration of breastfeeding of Mesolithic and Neolithic mothers is still scant, and second, the archaeological evidence of the role of new agricultural food in Neolithic weaning practices is even more incomplete than skeletal evidence. In other words, it is still not known whether the duration of breastfeeding became shorter with the Neolithic, and if so, what was the role of new weaning practices to this eventual reduction of the lactation period.

Considering skeletal evidence, recent methodological improvements in the resolution of stable isotope signals enabled reconstructions of infant (breast)feeding and weaning practices, which provide indirect evidence for the duration of lactation [20, 21]. In her review of isotopic studies on weaning in archaeological populations, Howcroft stated that ‘although the modal duration of breastfeeding was observed to be shorter in agricultural than in hunter-gatherer populations, the overall range of ages reported was similar’ [19]. And, as it was concluded by the same author, there are still many limitations of this type of study, for example the data for hunter-gatherer populations were based only on a few individuals. Also, the results of few studies which included both hunter-gatherer and agricultural populations give different impressions. Some of them found that the adoption of agriculture was not associated with a reduction of the duration of breastfeeding [22, 23]. On the other hand, a recent study in which the age at the start of weaning was assessed for 25 children individuals from prehistoric sites in the Central Balkans demonstrated two different feeding strategies by Mesolithic and Neolithic mothers, where the latter opted for a shorter breastfeeding period [24, 25]. These results indicate that the ‘weaning foods” hypothesis should not be rejected before more research is done on Early Neolithic and Mesolithic children in order to reconstruct characteristics of breastfeeding and weaning strategies during the process of Neolithisation.

And, although the current results of isotopic studies on the differences in the duration of lactation between Mesolithic and Neolithic mothers are inconclusive, we can assume that the Neolithic brought important innovations in weaning practices due to the availability of animal milk and cereal-based gruels. Given that prior to agricultural economies no infant could have been fed with that kind of food, which is widely used in infant feeding up to date, the Neolithic can be described as a defining moment in the evolution of human infant feeding practices–as ‘an infant feeding revolution’ [19]. However, probably due to the lack of investigation of prehistoric motherhood, weaning as an important part of motherhood remains unstudied by archaeologists, although it is reasonable to assume that there should be traces left after preparation, storage and serving of weaning food. Evan artefacts that could have been related to infant feeding, such as small spoons, remain unrecognized as a possible ‘weaning equipment’.

**Fig 1:**
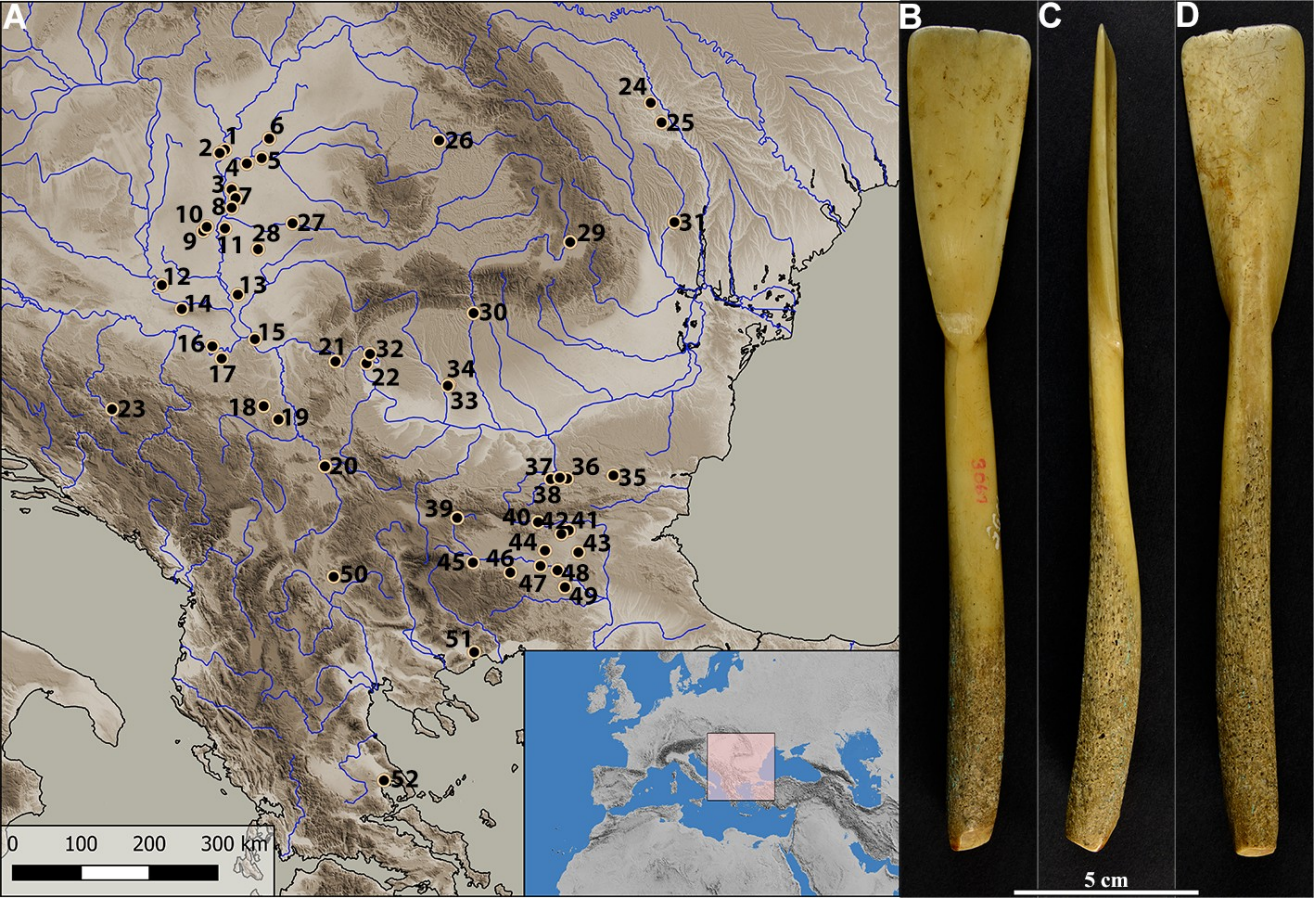
Bone spoons. (A) The distribution of Early Neolithic ‘V’ based bone spoons at 52 sites in South East Europe (*26*, *29*): 1.Tiszaug–Tópart; 2. Tiszaug; 3. Vata Tanya; 4. Sarvas; 5. Endröd; 6. Ecsegfalva; 7. Zsoldos Tanya; 8. Maroslele Pana; 9. Ludaš_Budžak; 10. Nosa Győngypart; 11. Srpski Krstur; 12. Donja Branjevina; 13. Mužlja; 14. Golokut; 15. Starčevo; 16. Obrež; 17. Grabovac; 18. Divostin; 19. Tečić; 20. Bubanj; 21. Lepenski Vir; 22. Velesnica; 23. Obre I; 24. Glăvănești Vechi; 25. Valea Lupului; 26. Gura Bacului; 27. Arad; 28. Besenova Veche; 29. Leș; 30. Valea Răii; 31. Trestiana; 32. Schela Cladovei; 33. Cârcea-Hanuri; 34. Cârcea Viaducti; 35. Ovcharovo; 36. Malkata Peštera; 37. Golemata Peštera; 38. Samovodene; 39. Čavdar; 40. Kazanluk; 41. Karanovo; 42. Azmak; 43. Kovačevo; 44. Asmaška; 45. Kapitan Dimitrevo; 46. Muldava; 47. Yabalkovo; 48. Nova Nadezhda; 49. Delcevo; 50. Amzabegovo; 51. Dikili Tash; 52. Sesklo. (B, C, D) A typical bone spoon from the site Grad-Starčevo (Spoon No 03–1995) (S1 movie) in front (B), lateral (C) and back projection (D).

Given the great importance of prehistoric mothers and babies for the demographic success, the aim of this article is to underline the necessity to investigate the phenomena related to prehistoric motherhood from both the archaeological and bioarchaeological perspective. Also, we provide evidence that a widely used type of Neolithic artefact, the bone spoon, was part of the new weaning equipment, and demonstrate that the lack of research on prehistoric motherhood resulted in the failure to recognize archaeological artefacts related to infant care. An important part of the prehistoric weaning equipment were V-based bone spoons, which appeared in the Early Neolithic, especially in Anatolia and the Balkans [26–29] (Fig 1A).

The description of spoons as particular types of Neolithic tools, the explanation on how they were made, as well as their distribution was given by John Nandris, who was among the few authors who interpreted them as a proper spoon [26, 30]. Although, to date, a detailed microscopic study of traces of use has not been conducted, on the basis of intensive traces of use on almost all spoons, many authors consequently interpreted them as spatulas–tools used for scraping flour from querns [31], for making decoration on ceramics [32], in the production of clothing and the application of pigments made from plant materials to leather, or cosmetic tools [29].

Contrary to those interpretations, the small dimensions of spoons and presence of intensive traces of use led us to the assumption that they were used for feeding babies. Spoons were made from cattle metapodial bones, modified into a concave or flat bowl and a slightly curved handle (Fig 1B). Their dimensions are small–the usual length is around 15 cm and the width of the bowl is from 2 to 2.5 cm [26]. We argue that they were used for feeding babies and that marks on them can be connected to the usual mouthing behaviour of children who may, up to four years of age, mouth objects up to 50 times during one hour [33]. Mouthing includes different activities, such as biting, nibbling, gnawing and pulling, which could all leave traces on the spoons. These activities are particularly frequent among children during the weaning period, when food other than breast milk is introduced into the diet. Feeding in the weaning period is considered not only as transitional from milk to solids, but also transitional from sucking to chewing and biting [34]. Teething is another reason children mouth objects at this stage of development, as mouthing alleviates the pain and discomfort caused by teething [35]. In order to test the hypothesis that marks observed on Neolithic bone spoons are tooth marks made by children, we developed two main test criteria: 1. the marks found on Neolithic bone spoons need to correspond metrically and morphologically to the experimental marks we produced by deciduous teeth on cattle bones; 2. the marks found on spoons are specific to these types of artefacts and are different from marks we analysed on other classes of Neolithic bone tools that were not used in any activity involving biting and gnawing by children (for example, awls). In addition to the first two criteria, we also hypothesize that the distribution of marks on the spoons is specific and bite-related and we expect to find marks in similar locations on both sides of spoons (front and back), as in most cases spoon would be in occlusion on both sides for each individual bite. Given the manner in which the spoons would have been used, we would also expect to find a greater frequency of marks in the recipient area of the spoon rather than on the handle. The analysed spoons originate from the Early Neolithic site of Grad-Starčevo in Serbia, the eponymous site of the Starčevo-Körös-Criş cultural complex, occupied for ~ 350 years from ~ 5800 to ~5450 cal BC.

## Materials and methods

The material used in this study consists of archaeological artefacts, used in the study of teeth marks, as well as primary teeth and cattle metapodial bones used for experimental purposes. All primary teeth were collected with the consent of the patient and written consent signed by the parents or carers. The study protocol was approved by the Ethical Committee of the Dental Clinic of Vojvodina, date of approval August 15^th,^ 2017, Protocol N^o^127/17. Cattle metapodial bones were obtained from the slaughterhouse Big bul foods. The legal form of this company is Limited Liability Company, it meets HACCP and TUV standards, is licensed to export meat and meat products to EU countries, and their work follows European laws, from energy support in the technological process to the filtration of wastewater into thermal energy.

### Information on analyzed specimens

From the site Starčevo-Grad, following spoons were analyzed: spoon No. 03–3548; spoon No. 03–3549, and spoon No. 03–6901. All of the spoons are part of the Early Neolithic Collection, Department of Archaeology, at the National Museum in Belgrade, led by Andrej Starović. For all of the analyzed spoons, a permission from the National museum in Belgrade, signed on 02.02.2017, was obtained. The institution did not provide the permission number.

From the site Vinča-Belo Brdo, following bone tools were analyzed: an awl, with the inventory number EDM 24 (2008); an awl, with the inventory number EDM 28 (2009); an awl, with the inventory number EDM 387 (2005); an awl, with the inventory number EDM 467 (2004); an awl, with the inventory number EDM 238 (2009); an awl, with the inventory number EDM 64 (2004); an awl, with the inventory number EDM +386 (2005); a burnisher, with the inventory number EDM 33 (2008); a fragmented fish hook, with the inventory number EDM 260 (2009); a lure hook, with the inventory number EDM +158 (2006).

For all of the analyzed tools, a permission from the Belgrade City Museum, signed on 18.09.2019 and registered under the number 02 12/93, was obtained.

### Information on dated specimens

From the site Starčevo-Grad, four bone spoons were AMS dated: Inv. No. 22/42; Inv. No. 24/2919; Inv. No. 23/2917; Inv. No. 28/2916. All of the spoons are part of the Archaeological Collection at the National Museum in Pančevo. For all of the dated spoons, a permission from the National Museum in Pančevo, signed on 21.03.2016, was obtained. The institution did not provide the permission number.

From the site Donja Branjevina, six bone spoons were dated: DBR 319; DBR 118; DBR 318; DBR 117; DBR 156, and DBR 159. All of the spoons are part of the Archaeological Collection at the Museum Unit in Odžaci. For all of the dated spoons, a permission from the Museum Unit in Odžaci, signed on 15.01.2016. and on 05.02.2016, was obtained. The institution did not provide the permission number.

### Archaeological background

The Early Neolithic site of Grad-Starčevo is situated on the left bank of the Danube in the Southern Carpathian basin, Serbia. The site (at c. 75 m above sea level) is located at the fringe of the modern village of Starčevo, 9 km south-east from Pančevo near Belgrade. The excavation of the site started in 1928 [36]. During this small-scale excavation, seven pits and pit-dwellings were discovered. Findings such as painted pottery, unique at the time, attracted the attention of the international archaeological community. The joint Serbian−American excavations started in 1931, in the course of which two pit-dwellings (No. l and 2) were explored [37]. Major field campaigns were carried out in 1932 [38], when a sub-triangular trench was excavated with eight pits and pit-dwellings (No 3, 4, 5A, 5B, 6, 7, 8 and 9) (map in S1 Fig). Additional tranches were opened (A-A extension and B some 15 m to the north of the main excavation area), but the stratigraphy was here obscured and archaeological materials of a younger age were found in the lowermost layers, on top of the loess underlying the site [39]. As described by Clason (1980), four main levels were initially recognized as humus, subhumus, culture and pit level, later renamed to humus, level I, level II and level III. The pits could be recognized starting from level III and they extended into the loess [39]. Smaller campaigns were conducted in 1969. and 1970, and, more recently, rescue excavations were undertaken in 2003−2007 [40]. The settlement was probably occupied for approximately 350 years (cca 5800−5450 cal BC) (dates in S1 Fig), based on 17 radiocarbon dates [41].

Although the Grad-Starčevo site is only partially published, recent analysis of the bone industry by S. Vitezović [42] and the documentation of the Peabody Museum, where seven spoons were stored after the excavations in 1931, show that 50 spatula-spoons had been discovered during the first two campaigns (1928, 1931−32) and two more were accidental finds given to the excavators. In the case of 19 spoons connected to pit-dwellings, the archaeological context is clear; for the rest, the documentation was not informative enough to connect them with a precise context of discovery. During the 1928 campaign, four spoons were found in pit-dwelling 2 and one spoon in pit-dwelling 4; during 1931, four spoons were found in pit-dwelling 1, and 2 spoons in pit-dwelling 2; and during 1932, one spoon was found in pit-dwelling 3, five spoons in pit-dwelling 5A, and two spoons in pit-dwelling 6. Three spoons analysed within this study, curated in the National Museum in Belgrade, originated from pit-dwellings: spoons No. 03–3458 and 03–3549 from pit-dwelling 2 (field campaign 1928) and spoon No. 03–6901 from pit-dwelling 6 (field campaign 1932). Spoons from pit-dwelling 2 were found on the floor and spoon No. 03–6901 was discovered in the fill of pit-dwelling 6.

### Experimentally produced primary teeth marks on cattle bone

**Fig 2:**
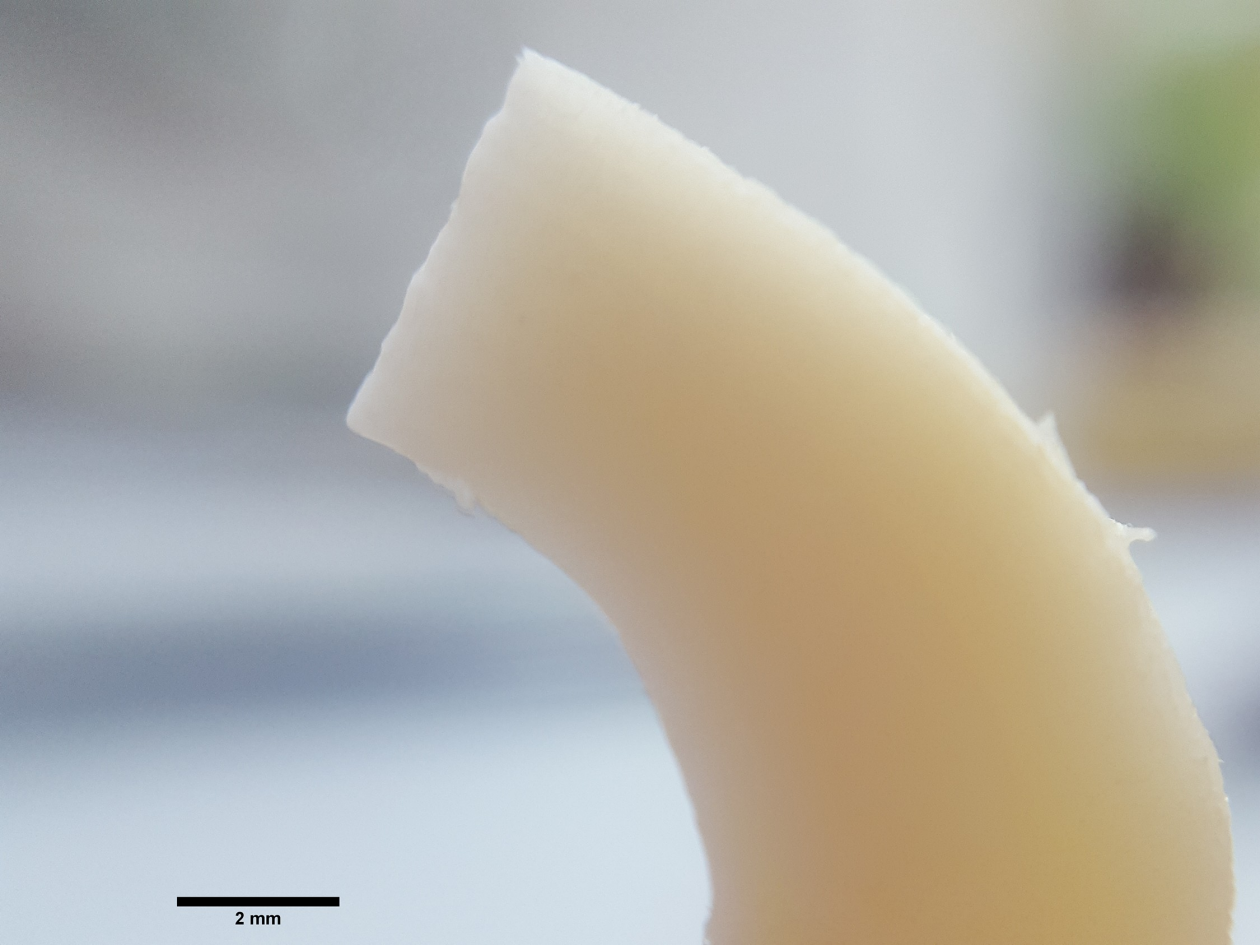
Cattle metapodial bones before chewing by primary teeth.

There has been an ongoing debate regarding bite-mark analysis between experienced forensics, anthropologists and odontologists, and, although there are many reports focusing on the way in which humans may leave visible tooth marks [43], experimental studies are still rare [44, 45]. Tooth marks are usually analysed following the methodology of Andrews and Fernández-Jalvo [46], and classified as punctures on bone surface, gnawing on bone surface, punctures on articular surfaces, punctures on spiral breaks, punctures on transverse breaks, punctures on split shafts, molar puncture prints made by multi-cusped teeth, punctures on intact bone edges and punctures on crenulated edges. Despite the observation of diversity of tooth marks, some studies focus on two specific types of tooth marks, pits and scores [47]–the marks that also appear to be most commonly present in our experimental study. Additional data exist about the dimensions of chewing marks produced by permanent human teeth [43, 48]. In our study, we hypothesize that marks on spoons were produced by primary teeth and in the literature, to the best of our knowledge, the description of possible bone surface bite marks inflicted by primary teeth has never been given. Thus, it was necessary to conduct an experimental study in order to identify the mark patterns human primary teeth leave on cattle bone surface with the force that children are capable of generating during eating and mouthing behaviour. This experiment, in which we produced 3151 primary teeth marks on cattle bone (Fig 2), provides morphological and metrical data, which we compared with marks detected on Neolithic spoons and other types of Neolithic tools.

**Fig 3:**
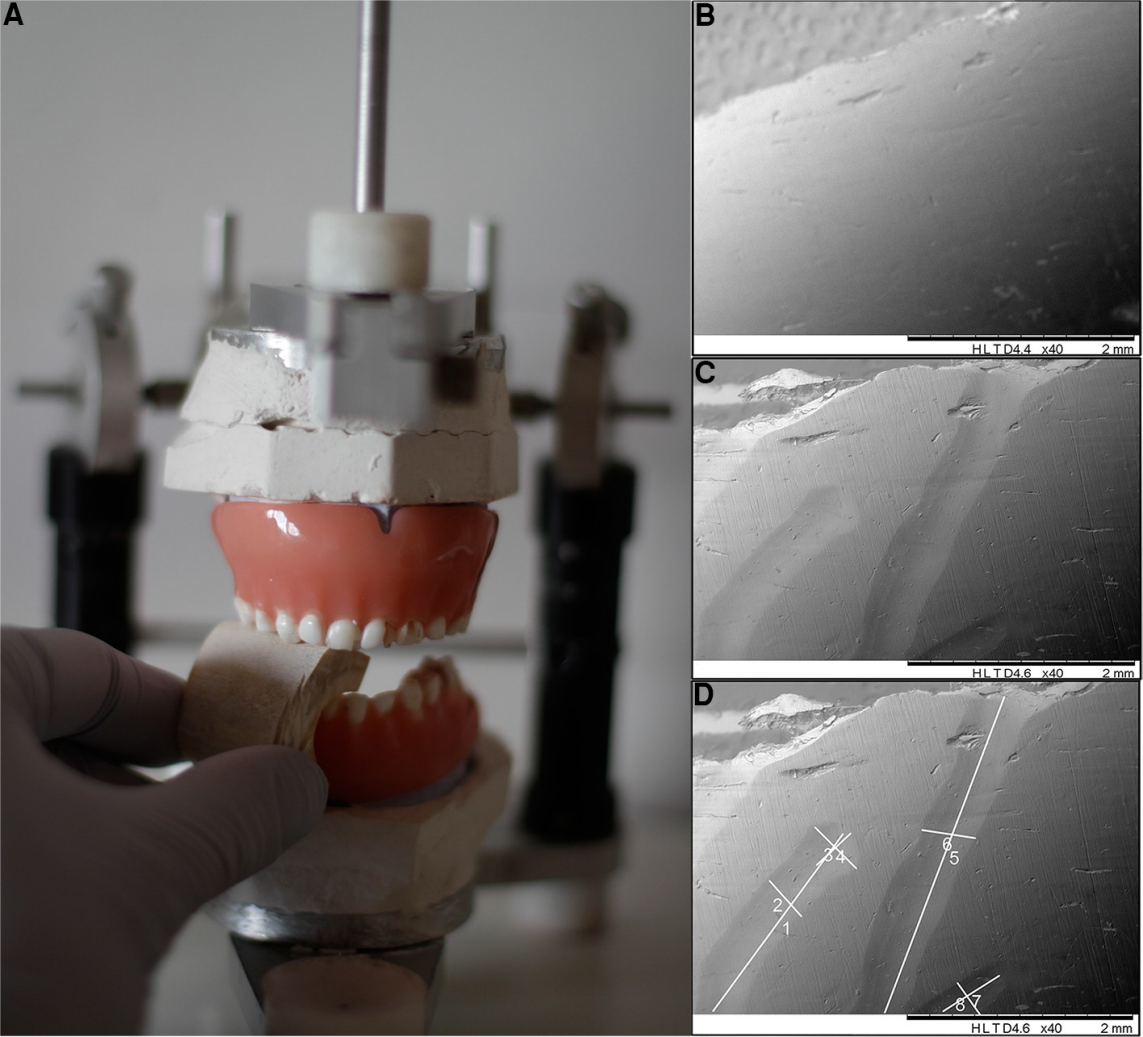
The experimental set-up. (A) The dental articulator, with primary teeth placed in casts of maxillary and mandibular gums constructed for a simulation of chewing; (B-D). The documentation of the experimental biting procedure, tooth M3; (B) Cattle metatarsal photographed before the experiment and (C) after the experiment; (D) Raw referent images obtained from the microscopes were processed and all measurements performed in Gwyddion open source software.

In order to analyse the metrical and morphological primary teeth marks on cattle bone, an experimental setup has been designed using natural donor intact primary teeth placed in partial and complete dentures (Fig 3A). Stone cast model of a child aged 3 were used as the template for the set of extracted natural donor primary teeth and for the fabrication of removable dentures. The procedure of denture fabrication followed the principles of conventional prosthodontic technique, but with a few modifications. The teeth on the study models were cut off and shellac base plates were adapted to upper and lower casts, and wax occlusal rims were then prepared following the same principles described in our recent report [25]. Working casts were mounted in a simple semiadjustable articulator. After the maxillary and mandibular casts were secured to the articulator with a Cdental stone, the vertical dimension screw was set, and the teeth were positioned in maximal intercuspation. The natural teeth were arranged on the wax occlusal rims with spacing that simulates the natural spacing expected at the age of 3. The waxed dentures were processed with heat cure acrylic resin. Experimental teeth were placed in two pairs of total dental prostheses, one pair of complete dentures and one pair of partial dentures with incisor and canines and the chewing simulation was performed in the articulator. In addition, six single representative teeth (maxillary incisor I, maxillary canine C, mandibular second molar M2, maxillary second molar M1, maxillary first molar M3 and mandibular first molar M4) were mounted into acrylic blocks 1cm x 1cm x 1cm, and the chewing simulation was performed using direct hand pressure.

Primary teeth of typical morphology were used in the experiment. Regarding central maxillary incisors, teeth with a entirely straight incisal edge and protruding cingulum were chosen. Second maxillary incisors with comparable morphological characteristics as central, but proportionally smaller and rounded distoincisal edge werechosen. All maxillary canines had well-defined, sharp and pronounced cusps. All first maxillary molars were with three cusps, two buccal and one lingual, with a protruding mesiobuccal cervical bulge. The second maxillary molars had four defined cusps. The mandibular central incisors were symmetrically flat when observed from buccal aspect, with cingulum existing on lingual surface. The second mandibular incisors had similar morphological characteristics, with incisal edges sloping toward distal part of the tooth and a more rounded distoincisal edge. The maxillary canines had a pronounced cusp, with the crown that was shorter and narrower labiolingually. The maxillary first molars had four cusps, a pronounced mesiobuccal bulge and a defined transversal ridge. The second maxillary molarswere with five cusps.

The tests were conducted on a fresh cattle metatarsal obtained from a local slaughterhouse. The cortical bone from the diaphysis was cut into 47 3 mm-thick slices with a water-cooled diamond-impregnated low-speed saw (Isomet Low-Speed Saw, Buehler; Lake Bluff, IL, USA). After the removal of the marrow with a water jet, the slices were stored at room temperature. They were polished before testing, using motorized silicon carbide discs of various grit sizes (200, 600 and 1200). The final preforms had average dimensions of 25mmx10mmx2mm. After the preparation, all preforms were photographed before the experiment from both sides by a scanning electron microscope (TM3030, Hitachi, Tokyo, Japan) and a Zeiss stereomicroscope at magnifications of 20X to 50X.

**Fig 4:**
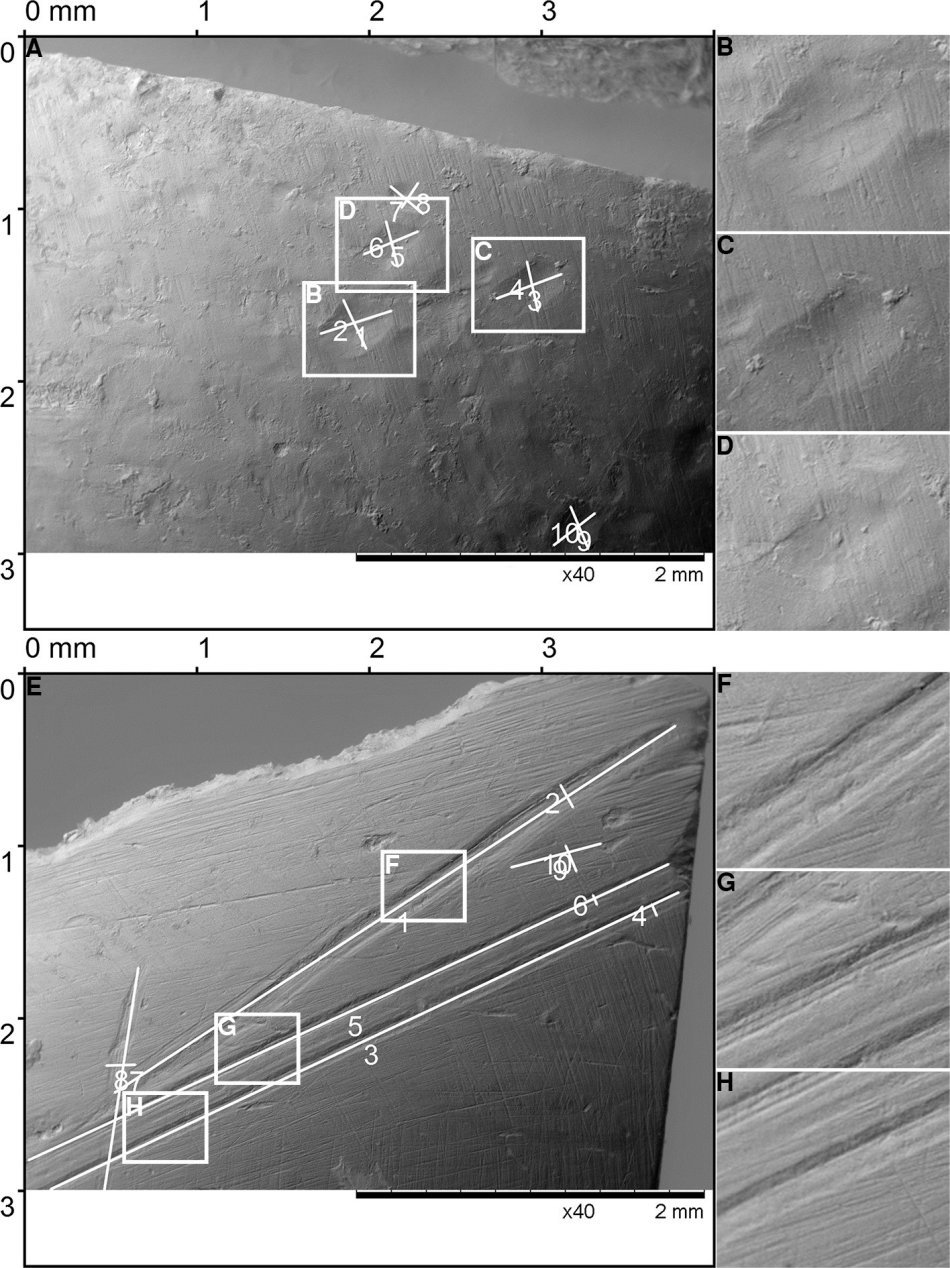
Teeth marks experimentally produced on cattle bone by incisors. (A) The pit marks produced by the ‘biting’ movement, details of the pit marks (B-D). (E) The score marks produced by the ‘tearing’ movement, details of the score marks (F-H).

**Fig 5:**
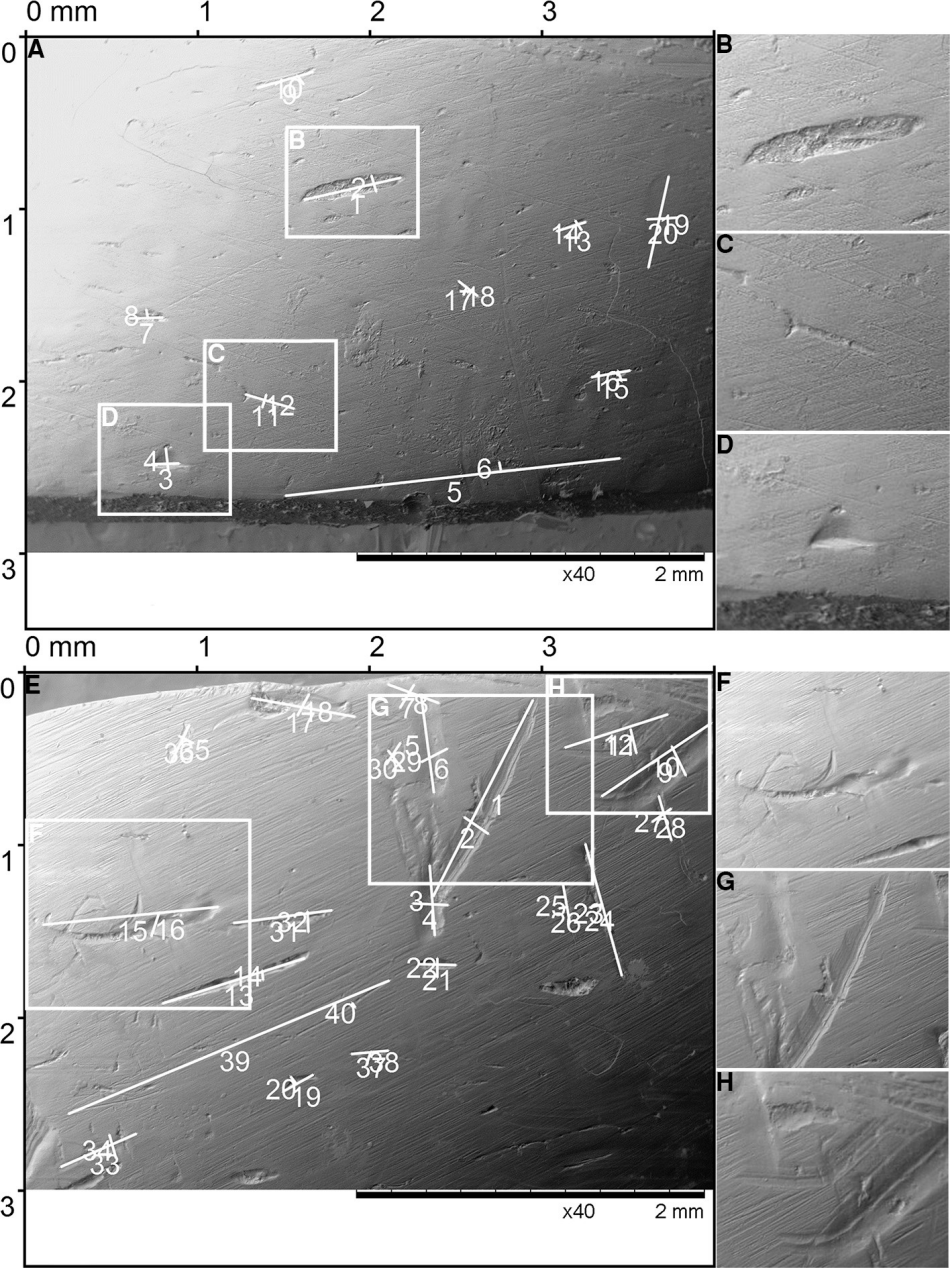
Teeth marks experimentally produced on cattle bone by canines. (A) The pit marks produced by the ‘biting’ movement, details of the pit marks (B-D). (E) The score marks produced by the ‘tearing’ movement, details of the score marks (F-H).

**Fig 6:**
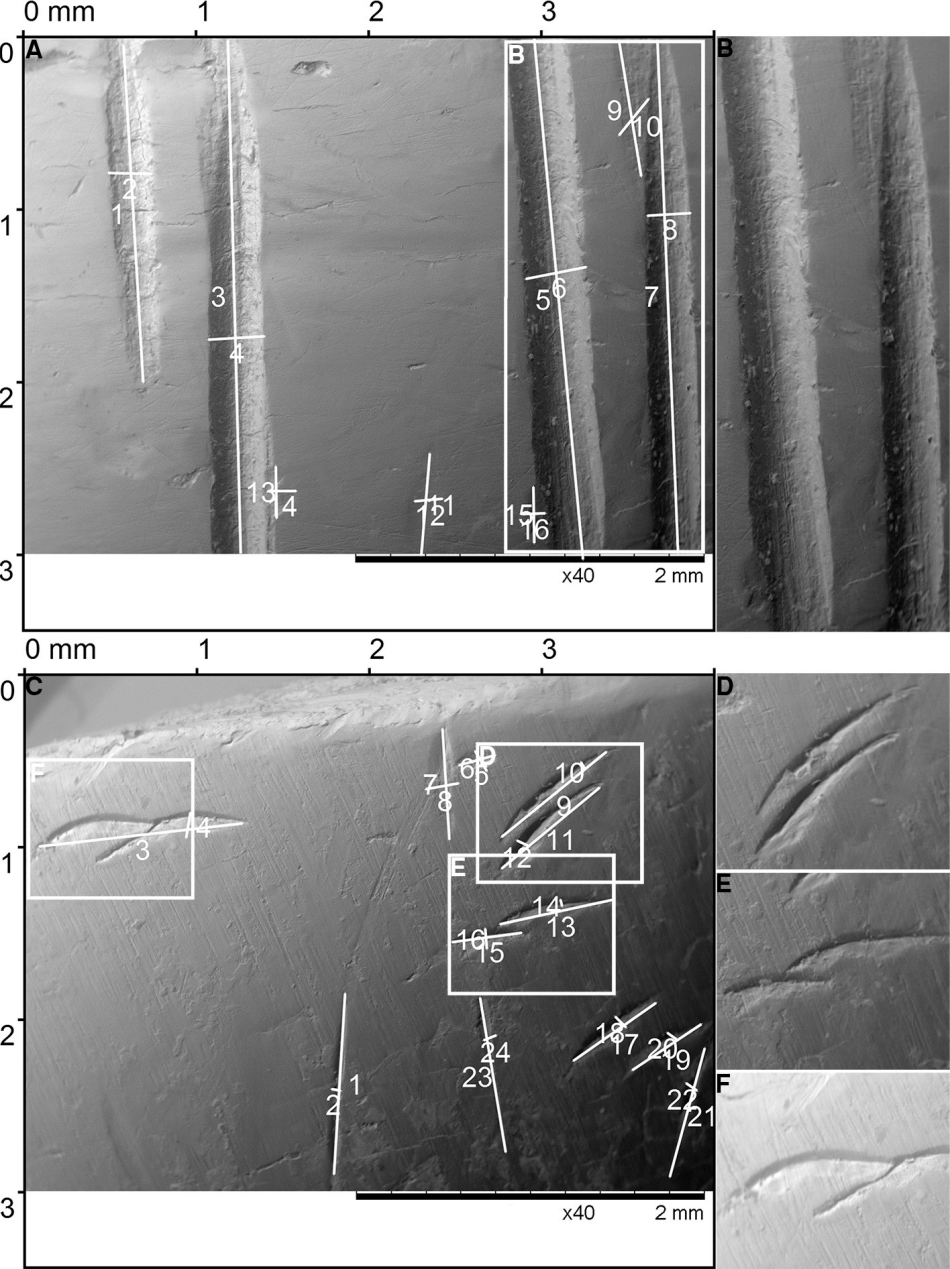
Teeth marks experimentally produced on cattle bone by the molar 1 and molar 2. (A) The score marks produced by the ‘tearing’ movement of M1, the details of the score marks (B). (C) The score marks produced by the ‘tearing’ movement of M2, the details of score marks (D-F).

**Fig 7:**
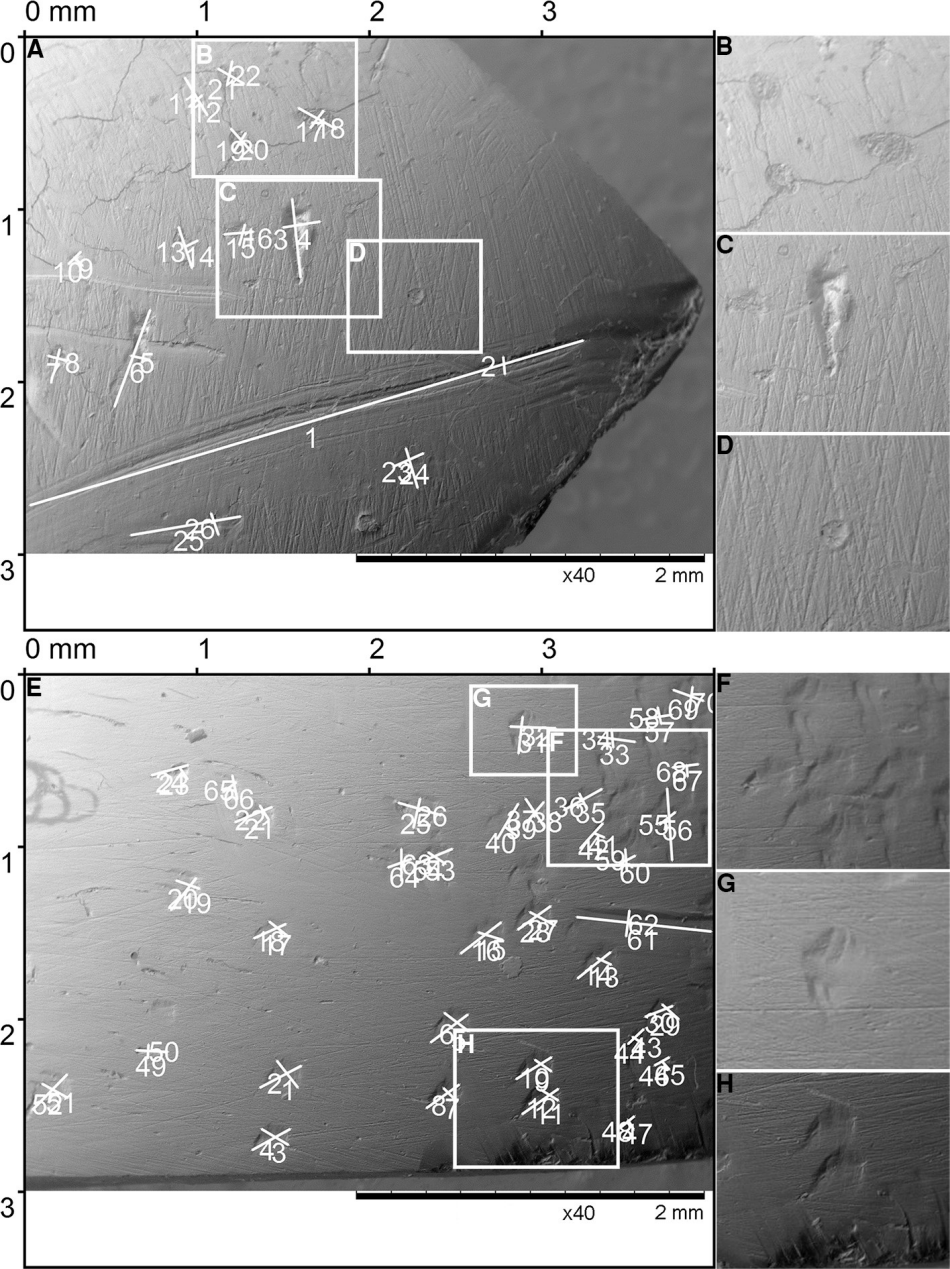
Teeth marks experimentally produced on cattle bone by the molar 3 and molar 4. (A) The pit marks produced by the ‘biting’ movement of the M3, details of the pit marks (B-D). (E) The pit marks produced by the ‘biting’ movement of the M4, details of the pit marks (F-H).

For each individual mark obtained during the experiment, the specific parameters were recorded in consistence with two recent reports [49,25]:

- referent image; tooth-mark identification has been accomplished by comparison of the bone sample images before and after the experiment (Fig 3B–3D). Bone samples were photographed using a Scanning electron microscope (SEM) or a Zeiss stereomicroscope. The SEM evaluation was conducted using TOPO SEM mode with constatnt magnification at 40X, while observations on the Zeiss stereomicroscope were performed using various magnifications from 20X to 50X. Data regarding the magnification, scale bar and mode were available for each referent image.
- Specimen numeration: all 47 specimens were marked numerically in order to facilitate the tracking of the relationship between the teeth used for the particular sample, intensity applied and the mechanism of the mark formation.
- Tooth type: depending on the tooth predominantly or exclusively used in the mark formation, the following designation was attributed to each mark: I-incisors (Fig 4A–4H), C-canines, (Fig 5A–5H) M1-maxillary second molar (Fig 6A and 6B), M2-mandibular second molar (Fig 6C and 6D), M3-maxillary first molar (Fig 7A–7D), M4-mandibular first molar (Fig 7E–7H).

- Mark mechanism: the mark mechanism was designated as ‘biting’ if the vertical movements in the articulator or by a single tooth were predominantly employed during the chewing simulation, and as ‘tearing’ if both the vertical and horizontal forces were used at the same time, that is, if the bone sample was pulled horizontally back or forth from the bite position.
- Intensity level: the force is classified as weak if it was up to 10N, moderate if it ranged from 10N to 100N, and strong if it was in the range of 100N to 200N. Five volunteers were included in the experiment under the supervision of BP. Two of the participants were fifth-year students of dentistry, three work as specialists in paedodontics. The participants were directed how to simulate chewing, and all of them were familiar with the aim of the experiment. The initial calibration regarding the applied force was performed using weights and scales, and the intensity level was provisionally defined and agreed by consensus. Complementary to the experiment results, all participants were asked to write down observations during the chewing simulation in order to identify the teeth which were predominantly or exclusively used in the chewing simulation, the mechanism of mark formation and the intensity level.
- Mark dimensions. The length, breadth and the relationship between the length and breadth (L/B ratio) of each mark were recorded. Raw referent images obtained from the microscopes were processed and all measurements performed in Gwyddion open source software (Fig 2D).
- Mark type: all marks with lengths less than four times their breadths were described as pits. Based on the shape in outline, the pits were classified into five groups 1) round–curved, circular and without sharp angles; 2) elliptical–elliptic, i.e. oval; 3) crescent–thin, curved, thick in the middle and tapering at each end; 4) drop-shaped–shaped like a drop of a thin liquid, globular at the bottom, tapering to a point at the top; 5) other–all other, angular and irregular shapes. Scores were defined as marks with lengths four times their breadths and longer. When measuring the breadth of a score, the narrowest distance along the linear groove was taken as the representative measurement. Additional characteristics of the scores were recorded as follows: the location of scores, the direction and frequency (multiple or single) of scores on each bone specimen, the presence of branching (branched or unbranched) and the shape of each score (straight or curved). The same criteria were used for mark evaluation on the three bone spoons and other classes of Neolithic tools, excluding tooth type, mark mechanism and intensity level.

The growth related development of bite, masticatory and mouthing movements and the occlusion forces which are generated during these functions and activities in infancy have not been clearly explained. It has been described that the vertical mandible movements described as munching begin around the sixth month, in the period before the primary first molar teeth erupt. Consequently, the horizontal mandibular movements during chewing and mouthing begin at the about the same time when second primary molars erupt, which is at the age of around 24 months. Finally, the complete rotary masticatory movements with constant occlusal contacts begin from around 3 years of age, and only after that child and adult masticatory functions and movements are similar [50–54]. As described by Scot and Halcrow [55], before the age of two years, vertical mandibular movements only produce crushing characteristics on occlusal surfaces on first primary molars of infants younger than two years of age, with certain sliding movements between teeth occurring with the introduction of horizontal movements of infants older than approximately 23 months. For this reason, we included single teeth bite marks with 6 representative teeth in this manner to simulate vertical movement.

### The recording of the marks on three Neolithic bone spoons from Grad-Starčevo

**Fig 8:**
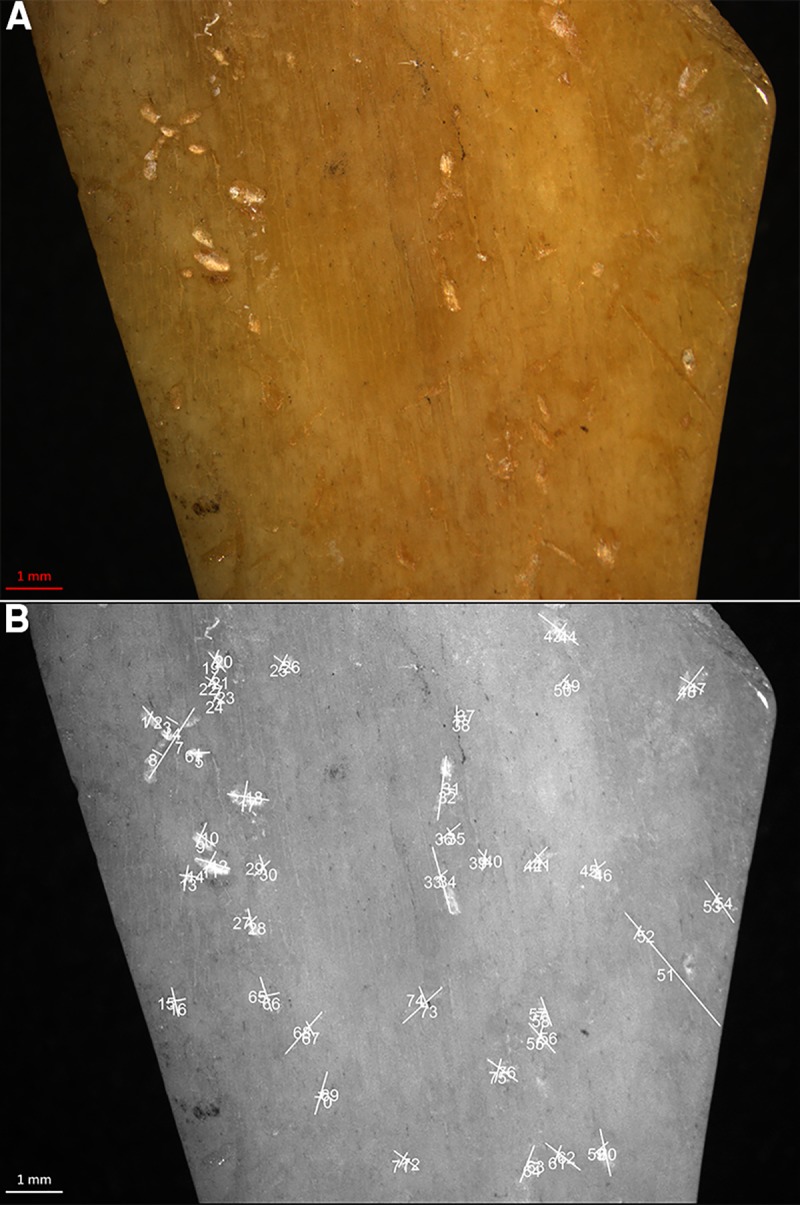
The recording procedure for marks on spoons. (A, B) An example of the procedure for recording and measuring of the marks on the spoons, grid 03–3549_FFA-C_5–6 before (A) and after measuring (B).

The same procedure of recording was used for the marks on Neolithic spoons (Fig 8A and 8B) and on the bone tools from the site of Vinča-Belo Brdo.

**Fig 9:**
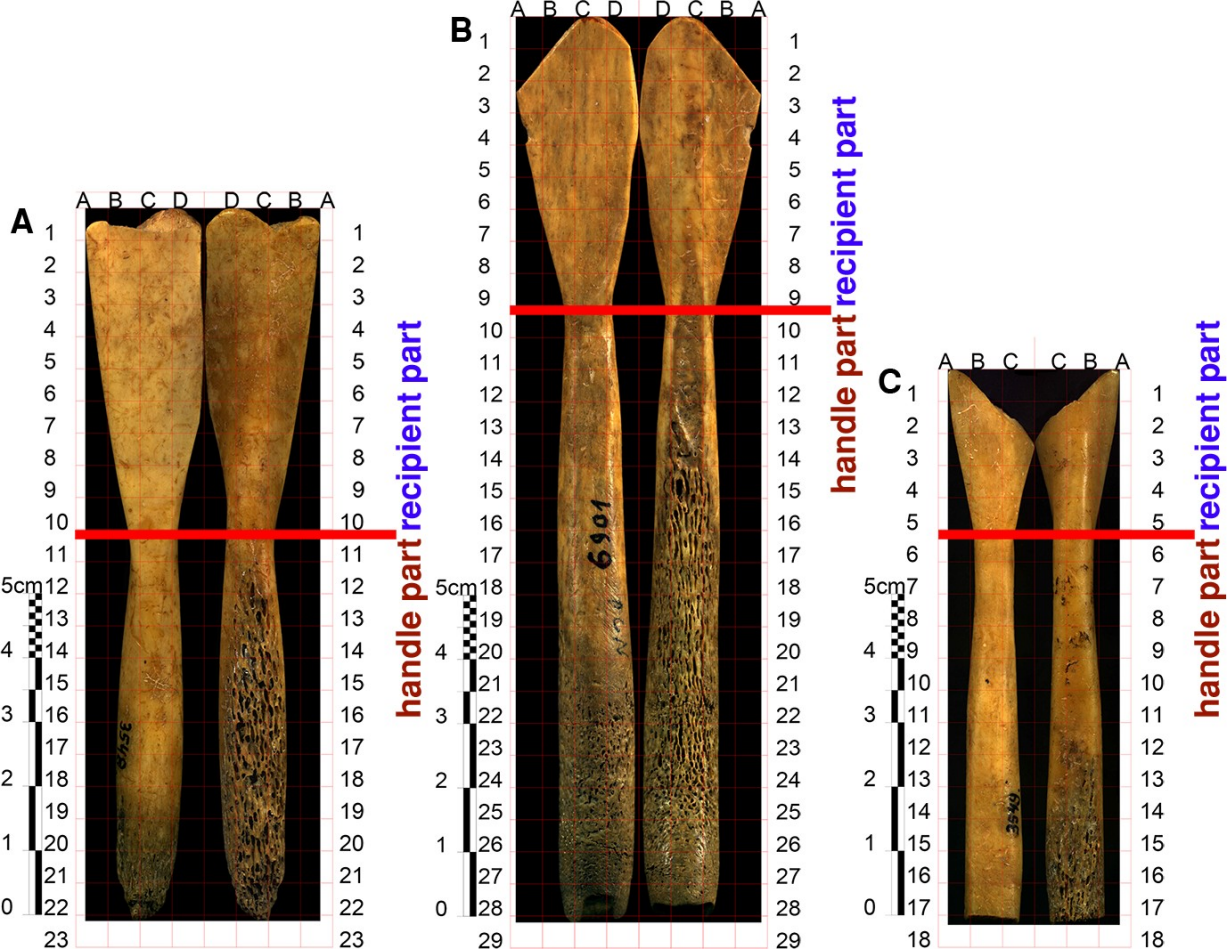
The division of spoons into quadrants and parts. The quadrants on the spoon 03–3548 (A), merged quadrants for double sided analysis: A7+B7; D11+C11; A6+B6; D20+C20; D12+C12; D10+C10; D13+C13; D14+C14; 03–6901 (B), merged quadrants for double sided analysis: A2+B2; A6+B6; A7+B7; B1+C1; C10+D10; C11+D11; C9+D9; 03–3549 (C), merged quadrants for double sided analysis: A5+B5; A13+B13; A14+B14; A4+B4; 5 B1+B2.

Additionally, the analysis of the marks on Neolithic spoons included the creation of a gridded view of both sides of the spoons, for which we produced a composite image out of, on average, 110 captures per side (Fig 9). Images were acquired by Zeiss Stemi 508, with an AxioCam ERc 5s camera attached to Stemi 305 trino microscope body, and 0.5x FWD 185 mm front optics.

In the image production, we aimed for the overlap of front and side views to be higher than 60%. Acquired images were uploaded to the Microsoft Image Composite Editor (ICE), creating a seamless high-resolution rendering of the entire surface of the artefact. This composite view was imported into AutoCAD 2014 and scaled to accurate dimensions, which was followed by the introduction of a control grid with cells 5X5mm in size. In the grid, Y-axis is labelled with integer values, ascending from top to bottom of the image. The axis drawn through X0 represents the line separating the two views of the spoon, where the front is on the left and the back is shown on the right. The X-axis is labelled using letters listed in the order that reflects the order of the cells of the grid laid over the surface of the artefact: i.e. the front side is labelled with letters in ascending order from left to right, while the back side is labelled in ascending order from right to left. In such a way, grid cells labelled A1, for example, show the front and the back of the same section of the artefact. This grid was later used for another session of image acquisition–this time, the images were acquired without an extended overlap, and using the grid numbering system for guidance.

## Results

**Fig 10:**
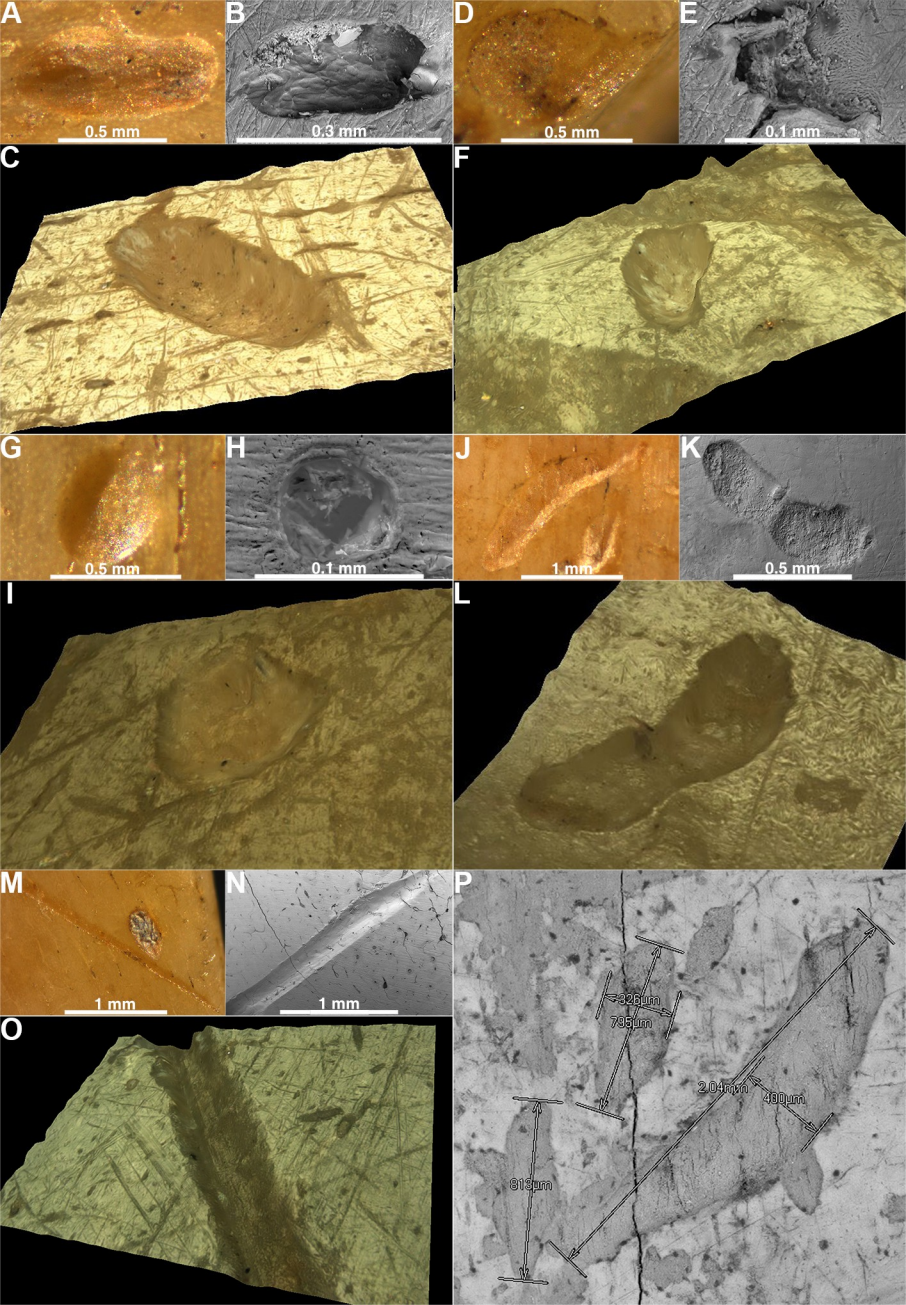
The pits and scores from the experimental study and the spoons. An elliptical pit–a Neolithic spoon (A), the experimental study (B), a 3D pit from a spoon (C); a drop like pit—a Neolithic spoon (D), the experimental study (E), a 3D pit from a spoon (F); a round pit–a Neolithic spoon (G), the experimental study (H), a 3D pit from a spoon (I); a crescent pit–a Neolithic spoon (J), the experimental study (K), a 3D pit from a spoon (L); a score—a Neolithic spoon (M), the experimental study (N), a 3D score from a spoon (O). (P) The measurements of mark dimensions—the length and breadth.

In our study, *first* we produced experimentally 3151 marks of primary incisors, canines and molars on a fresh cattle bone (data in S1 Dataset). Two specific mark types were detected: pits and scores. The pits appear in elliptical (Fig 10B), drop-like (Fig 10E), round (Fig 10H), crescent (Fig 10K) and irregular shapes. The breadth, length (Fig 3P) and the relationship between the length and breadth (the L/B ratio) were measured for each pit. The average length of experimentally produced pits is 0.45 mm, the average breadth is 0.23 mm, and the average L/B ratio is 2.12 mm. The scores produced by primary teeth appear as multiple or single, branched or unbranched, and in a straight or curved shape. The breadth was measured for each score, and the average breadth is 0.16 mm. The length of scores was not measured, as it depended more on the experimental set-up than the intrinsic properties of teeth marks.

### A comparison of experimentally produced primary teeth marks with the marks on Neolithic spoons and with marks on other types of Neolithic tools

**Fig 11:**
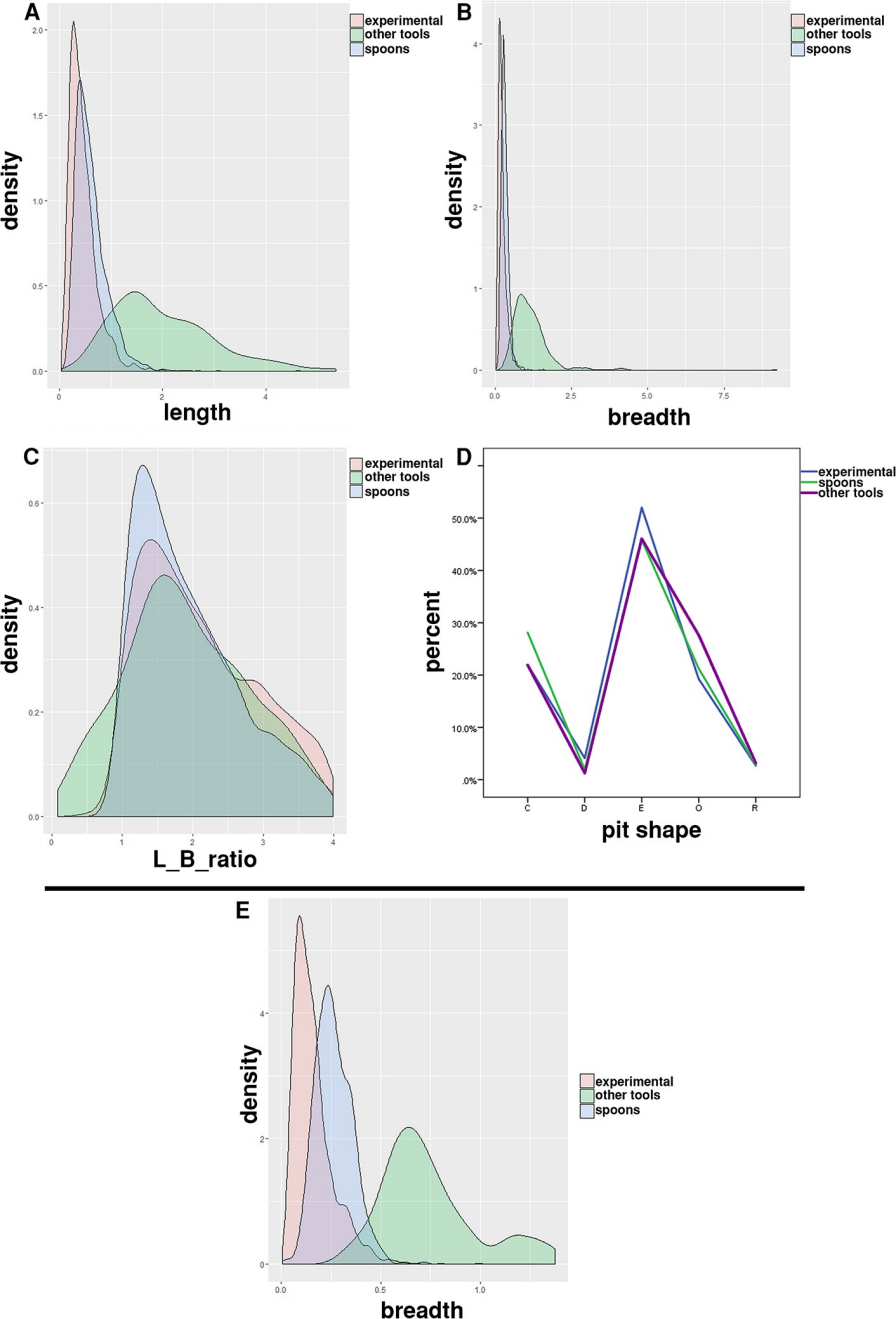
The comparison of marks. The comparison of size and shape distributions of experimental marks produced by primary teeth on cattle bones, the marks on Neolithic spoons and the marks recorded on other classes of Neolithic bone tools. The distributions of metric variables are presented as kernel density graphs. (A) The length of pit marks. (B) The breadth of pit marks. (C) The length to breadth ratio for pit marks. (D) Qualitatively defined shape categories. (E) The breadth of score marks.

The comparison showed that the range of values recorded on bone spoons is within the range of experimentally derived values. The full statistical correspondence of distributions was not expected, as the experimentally derived distribution is influenced by the specific combination of experimental factors, which does not have to, and probably does not correspond to the actual combination of these factors in the past. In addition, all or most of the shapes occurring in the archaeological material are also found in the experimental results. For their frequency distribution, the same limitation in statistical correspondence applies as to the metric variables. For the pits, the kernel density graphs were constructed in order to evaluate the degree of overlap in mark size (the length and breadth) and shape (the length to breadth ratio) between the experimental marks and those observed on spoons (Fig 11). The degree of overlap is substantial for all three variables, with the highest level of overlap in the L/B ratio (Fig 11C), meaning that the shape of archaeological and experimental pit marks is very similar. The range of values from spoon marks is within the range of values from the experiment. The breadths of the scores from spoons also overlap with the breadths of experimental marks (Fig 11E). As for pit shapes, all types were present in both groups in very similar relative frequencies (Fig 11D). For the comparison with marks on other bone tools, we chose ten tools from the Neolithic site Vinča-Belo Brdo − awls, burnishers and fishhooks, i.e. the tools on which the traces of primary teeth were not expected. The average density of marks on these bone tools is by an order of magnitude smaller than the mark density on the spoons: the mean density of marks on spoons is 0.345 marks/mm^2^ (range 0.253−0.361 marks/mm^2^), whereas the mean density of marks on other bone tools is 0.058 marks/mm^2^ (range 0.018−0.17 marks/mm^2^). The differences in the absolute frequencies of marks are also high, with spoons having 743.33 marks on the average (range 556−884), and other bone tools having 35.7 marks on the average (range 9−61).

The distributions of metric size variables for both pits (the length, breadth) and scores (the length) observed on other bone tools are apparently different from the distributions of corresponding variables of spoon marks (Fig 11). This indicates that different factors were responsible for their formation. Fig 11 clearly shows that the marks produced experimentally and those observed on spoons have more similar distributions of metric size variables in comparison to the marks on other bone tools.

However, there is a high degree of overlap in the length-to-breadth ratio for pit marks from all three classes (Fig 11C). In terms of shape, there are also no significant differences in frequency profiles between the experimental, spoon and other bone tool marks (Fig 11D). This indicates that, although the shapes of marks in the experimental, spoon and other bone tools are similar, their size is very different, with the experimental and spoon marks having smaller dimensions than the marks on other classes of bone tools.

### The distribution of marks on the spoons

In addition to the two main criteria, we also hypothesize that the following patterns should be observed regarding the distribution of marks on different parts of the spoon. We expect to find marks in similar locations on both sides of the spoons (front and back) as, in most cases, the spoon would have been in occlusion on both sides at each individual bite. There should also be no significant differences in the number of marks between the front and the back side of the spoon. Given the manner in which spoons would have been used, we would expect to find greater frequency of marks in the recipient area of the spoon than on the handle. Therefore, if the marks were made by teeth, we expect to find the following statistical patterns in the distribution of marks on the spoons:

1) A positive correlation between the number/density of marks on the front and the back side of the spoon.

2) No significant differences in the average density of marks between the front and the back side of the spoon.

3) A greater average density of marks in the recipient area of the spoon than on the handle.

In order to test whether the spoons fulfil these criteria, a grid of 10x10 mm quadrants was laid over each side of the spoon (Fig 9). For each quadrant, the number of marks was recorded. The quadrants were aggregated in two ways:

1) The quadrants were grouped into horizontal segments–e.g. the quadrants A1, B1, C1 and D1 formed segment 1, the quadrants A2, B2, C2, and D2 formed segment 2, and so on. The segment is the basic unit of observation. Only the segments where the marks were present on each side were used in the analysis. For each segment, the number of marks on the front and the back side was derived by summing the number of front and back marks from the quadrants that constitute the segment. The total area of the spoon surface encompassed by the segment was calculated by summing the areas of the constituent quadrants. Since the segments differ in the area they cover, we could not use the number of marks as a measure, as it would introduce false correlations due to the size effect. Therefore, we calculated the front and back density of marks for each segment by dividing the number of marks on the front and the back side, respectively, with the area of the segment. In this way, the differences between spoon surface areas included in the segments are accounted for. The spoon segments were grouped into the recipient or handle segments in respect to the spoon part that they cover. For example, spoon No. 03–3548 segments 1−10 are recipient segments, whereas the rest are handle segments (Fig 9).

2) The basic analytical units consist of two groups: a) the quadrants that fully cover the spoon surface area (e.g. the quadrant C3 in Fig 9)–‘full quadrants’; b) the merged quadrants: if the spoon surface area covered by a quadrant is less than 30% of the total quadrant area (~100 mm^2^)–‘a partial quadrant’–, then it is merged with the nearest ‘full quadrant’ to its right or left. If there are ‘partial quadrants’ on both sides of the ‘full quadrant’ (e.g. ‘the partial quadrants’ B11 and D11 flanking the ‘full quadrant’ C11), all three are merged into a single unit. The number of marks on each side and the spoon surface area are recorded for each unit. As the units differ in the spoon surface area that they cover (the merged quadrants have greater surface areas than the unmerged quadrants), we calculated the mark density value for each side of each unit by dividing the number of marks in a unit with its surface area, for the front and back side. Again, the units were divided into recipient and handle units.

A scatterplot was produced to visually evaluate whether there is a correlation between the number of marks on the two sides of each quadrant and the Pearson’s correlation coefficient was calculated to quantify the correlation (with a statistical test to evaluate its statistical significance). A positive and significant correlation would signal that the criterion was met. The paired t-test was used to test whether there are significant differences in the average mark density between the front and the back side of the spoon. The Mann−Whitney test was used to test for differences in density between the recipient and handle area.

**Fig 12:**
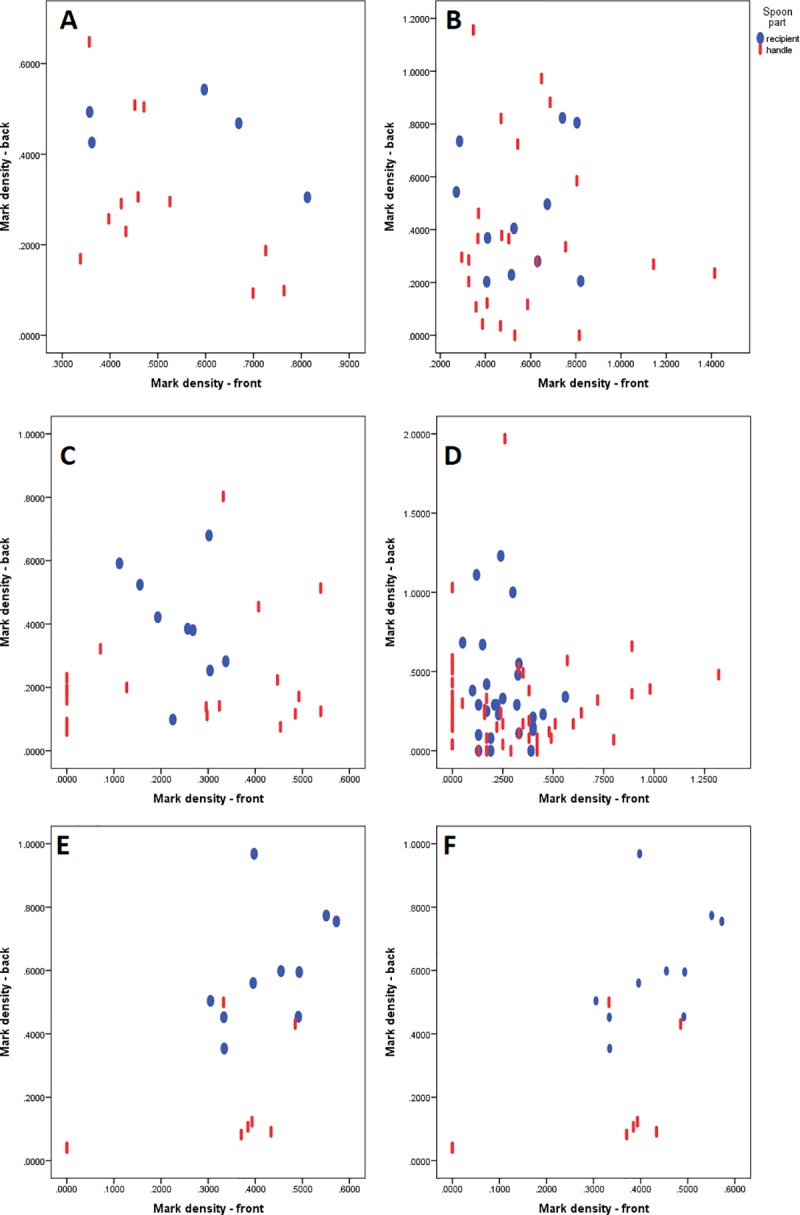
The front vs. back mark density on spoons. (A, B) Spoon No. 3549. Left panel: horizontal segments (A) right panel: merged and unmerged quadrants (B). (C, D) Spoon No. 6901. Left panel: horizontal segments (C), right panel: merged and unmerged quadrants (D). Spoon No. 3548. Left panel: horizontal segments (E), right panel: merged and unmerged quadrants (F).

**Spoon No. 03–3549.** 1) The scatterplot of mark densities of horizontal segments suggest that there is a negative relation between the front and the back mark density (Plot A in Fig 12). This is confirmed by the correlation coefficient, which is moderate but not significant (r = -0.385, p = 0.064, N = 17). There are significant differences in the mark density between the front and the back segments (t = -2.719, df = 16, p = 0.0075), with higher density on the front side. There are marginally significant differences in the median density of marks between the recipient and the handle segments, for the back (Mann−Whitney U = 14, exact p = 0.052) but not for the front side (Mann−Whitney U = 26, exact p = 0.361), with the recipient having the higher average density of marks on both sides of the spoon.

2) The scatterplot of mark densities of merged and unmerged quadrants (the second method of building observation units) (Plot B in Fig 12) suggests that there is no correlation between the front and the back mark density (r = -0.02, p = 0.454, N = 35). There are significant differences in the mark density between the front and the back segments (t = -2.425, df = 34, p = 0.0105). There are no significant differences in the median density of marks between the recipient and the handle segments, for both the front (Mann−Whitney U = 124, exact p = 0.394) and the back side (Mann−Whitney U = 100, exact p = 0.133).

**Spoon No. 03–6901.** 1) The scatterplot of mark densities of horizontal segments (Plot C in Fig 12) suggests that there is no correlation between the front and the back mark density (r = -0.098, p = 0.31, N = 28). There are no significant differences in the mark density between the front and the back segments (t = 0.726, df = 27, p = 0.237). There is a statistically significant difference in the median density of marks between the recipient and the handle segments, for the back (Mann−Whitney U = 36, exact p = 0.007) but not for the front side (Mann−Whitney U = 79, exact p = 0.392), with the recipient having the higher average density of marks on the back side of the spoon.

2) The scatterplot of mark densities of merged and unmerged quadrants (the second method of building observation units) (Plot D in Fig 12) suggests that there is no correlation between the front and back mark density (r = -0.01, p = 0.465, N = 78). There are no significant differences in the mark density between the front and the back segments (t = -0.634, df = 77, p = 0.264). There are no significant differences in the median density of marks between the recipient and the handle segments, for both the front (Mann−Whitney U = 673, exact p = 0.338) and the back side (Mann−Whitney U = 575.5, exact p = 0.119).

**Spoon No. 03–3548.** 1) The scatterplot of mark densities in horizontal segments suggests that there is a positive correlation between the front and the back mark density (Plot E in Fig 12). This is confirmed by the correlation coefficient, which is moderately high and significant (r = 0.585, p = 0.005, N = 18). There are no significant differences in the mark density between the front and the back segments (t = 0.727, df = 17, p = 0.238). There are significant differences in the median density of marks between the recipient and the handle segments, for both the front (Mann−Whitney U = 21, exact p = 0.048) and the back side (Mann−Whitney U = 4, exact p = 0.0003), with the recipient having the higher average density of marks on both sides of the spoon.

2) The scatterplot of mark densities of merged and unmerged quadrants (the second method of building observation units) suggests that there is a positive correlation between the front and the back mark density (Plot F in Fig 12). This is confirmed by the correlation coefficient, which is moderate and significant (r = 0.421, p = 0.001, N = 51). There are no significant differences in the mark density between the front and the back segments (t = 1.249, df = 50, p = 0.109). There are significant differences in the median density of marks between the recipient and the handle segments, for both the front (Mann−Whitney U = 188.5, exact p = 0.022) and the back side (Mann−Whitney U = 104, exact p = 0.00006), with the recipient having the higher average density of marks on both sides of the spoon.

**Fig 13:**
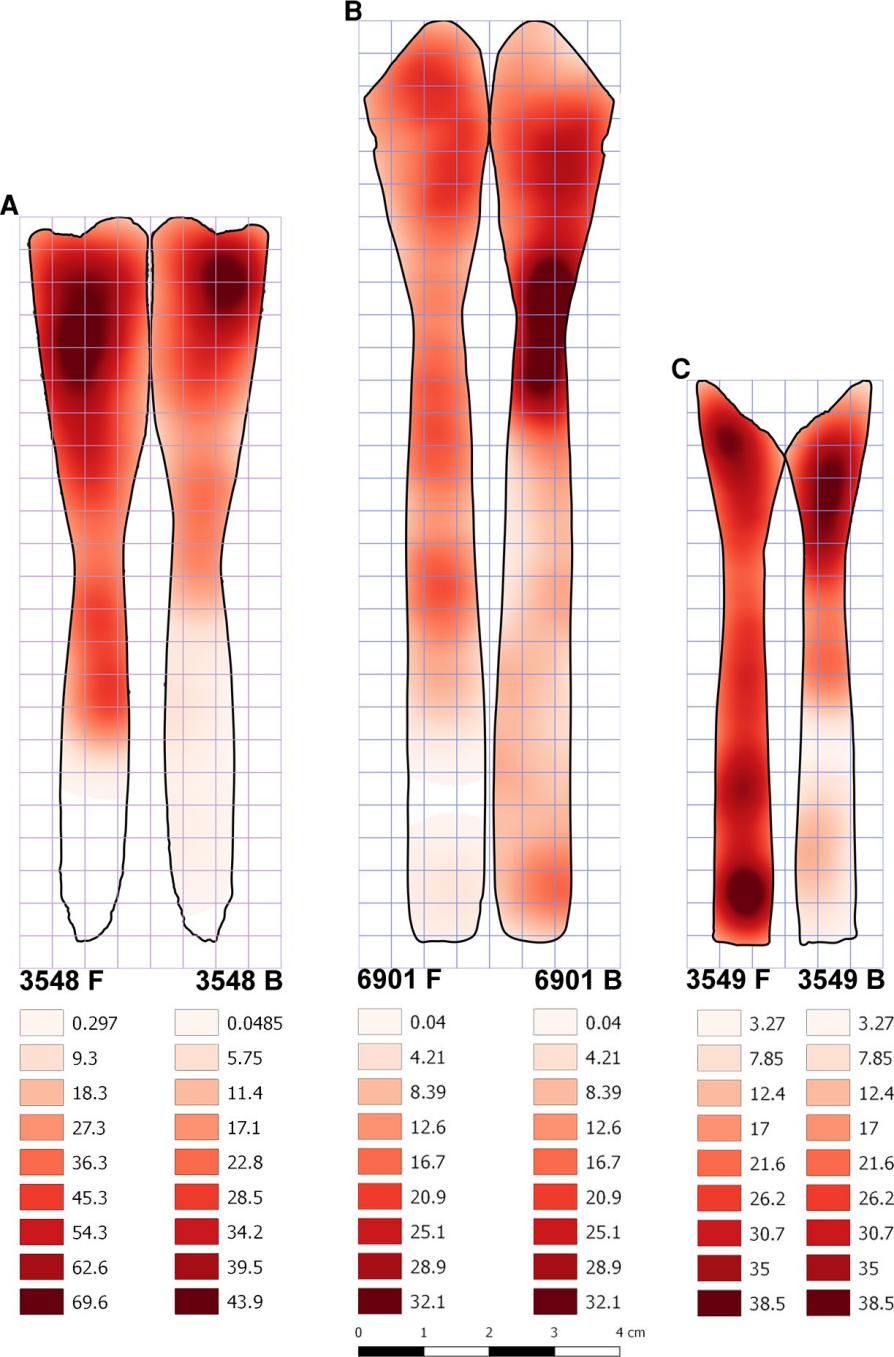
The density of marks (pits and scores) on the front and back sides of spoons. The schematic drawing of spoons from the Neolithic site Grad-Starčevo shows the distribution of marks (pits and scores) on the front and back side on the spoons No. 3548 (A), No. 6901 (B), No. 3549 (C). The general correspondence of the front and back areas of high and low density is apparent from the maps in Fig 13.

Let us summarize the results of the analysis of double-sideness of marks on spoons.The marks on the spoon No. 3548 confirm the expectations perfectly, as they show a clear correlation of the mark frequency between the front and the back side, that is, no major difference in the frequency of marks between the front and the back side, and a significantly higher frequency of marks on the recipient than on the handle (Fig 13A). The spoon No. 6901 (Fig 13B) partially conforms to this pattern as there are no differences in the average density of marks between the front and the back and the density of marks is higher on the recipient than on the handle, but this applies only to the back side of the spoon, and only for one kind of quadrant aggregation (into horizontal segments). The correlation in mark density between the two sides is absent on the spoon No. 6901. The spoon No. 3549 (Fig 13C) does not meet statistical expectations on the distribution, but it should be noted that a large portion of the recipient of this spoon is missing, which might have affected the results (e.g. the reduced range of mark density for correlation analysis).

*Second*, we analysed microscopically 2230 marks on three Neolithic spoons by applying the same measurement procedures as for the experimental marks. As in the experimental study, two types of marks appeared—pits (Fig 10A, 10C, 10D, 10F, 10G, 10I, 10J and 10L) and scores (Fig 10M and 10O). As we did not want to assume in advance which marks were made by human teeth, we decided to record all observable marks (greater than 100 microns) on spoon surfaces. Although this inevitably implies that at least some of the recorded marks were not human teeth marks, it would have been methodologically incorrect to record only marks that are consistent with the shape and metrics of marks derived in the experiment, as it would introduce strong confirmation bias in the analysis. If the marks were indeed made by children biting the spoon we should be able to show that most of the marks correspond metrically and morphologically to milk teeth marks and are different from marks usually found on bone tools that were certainly not used as spoons and were not subject to human biting.

**Fig 14:**
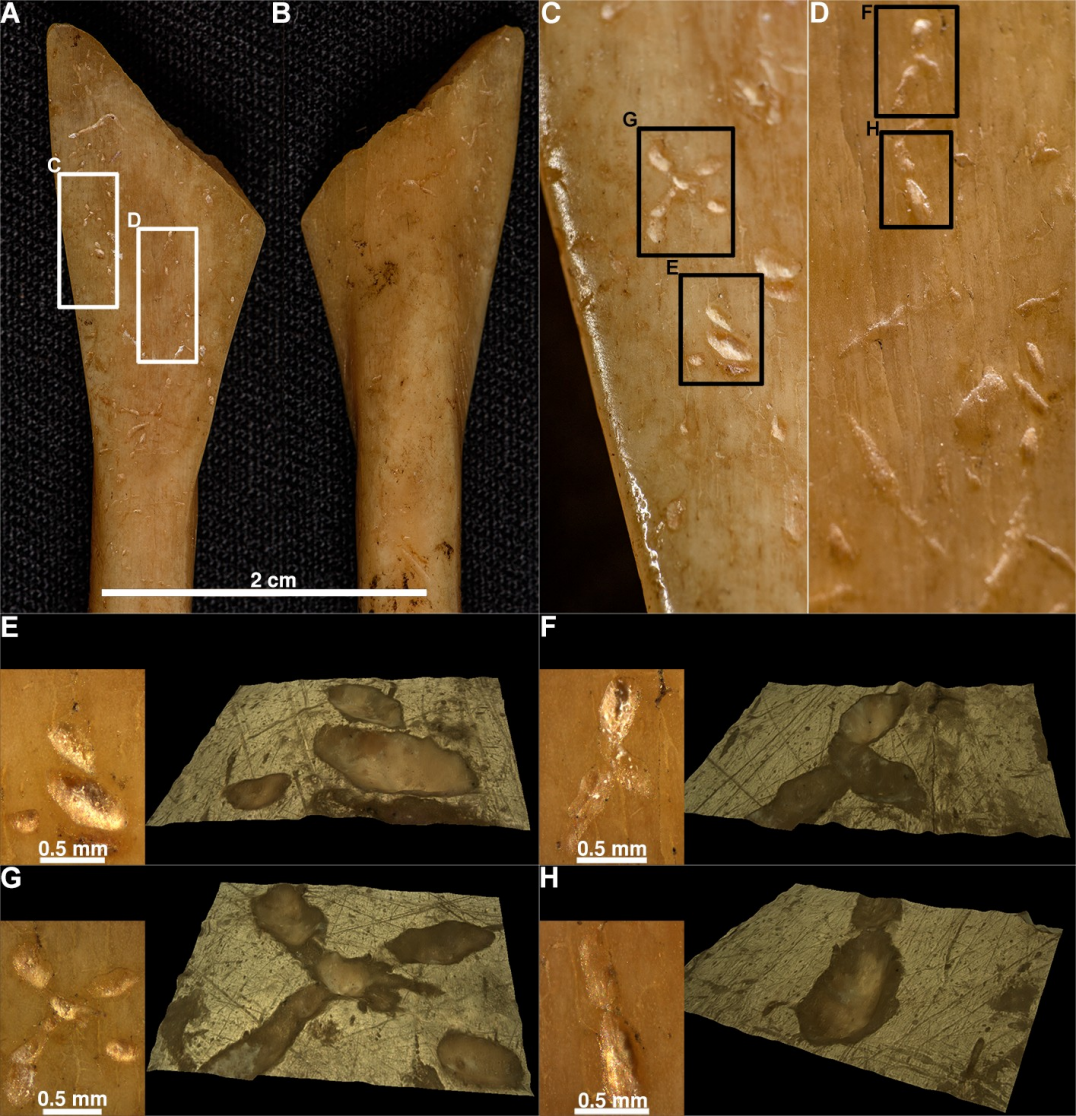
The marks on spoons from the site Grad-Starčevo. The Spoon 03–3548, front (A), back (B); the Spoon 03–6901, front (C), back (D). The Spoon 03–6901, front (C), back (D). The details of spoons with numerous pits and scores, the Spoon 03–3548 front (E, F) and spoon 03–6901, front (G) and back (H). A microscopic representation of single pits and scores (I-P): the Spoon 03–3548 three grouped pits (I), drop like pit (J, K), elliptical pit (M), round pit (N), score (O); spoon 6901, two elliptical pits (L), score (P).

**Fig 15:**
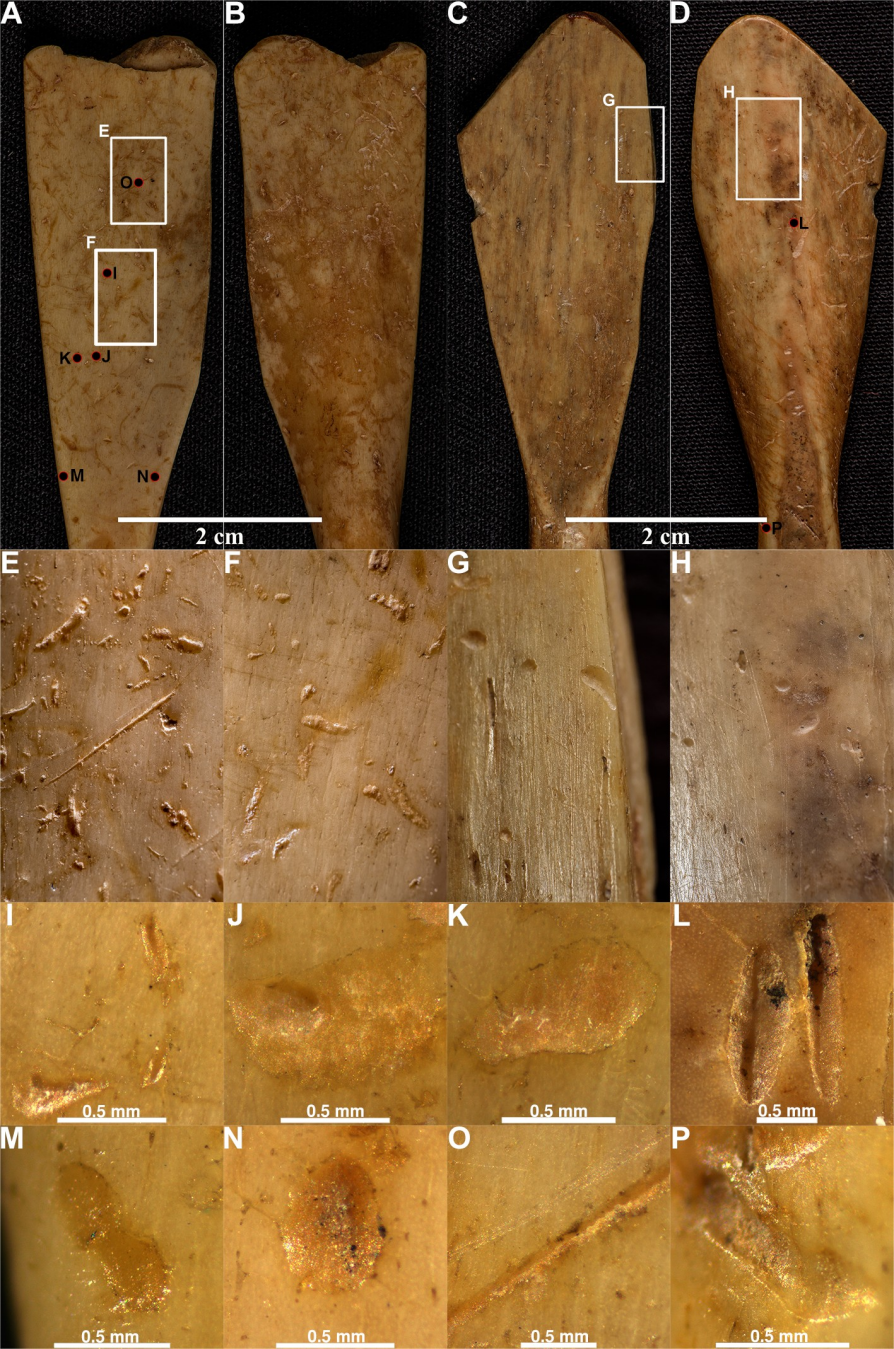
The marks on the Spoon No. 03–3549 from Grad-Starčevo site. (A, B) The Spoon 03–3549, front (A), back (B). (C, D) The details of the spoon with numerous pits and scores, front (C), back (D). (E, F, G, H) The microscopic and 3D microscopic representation of pits.

On the three analysed spoons from the Neolithic site of Grad-Starčevo, we detected 438 scores and 1792 pits. The mean length, breadth, and L/B ratio values for the pits are 0.61, 0.32, and 1.947 mm, respectively. The mean breadth of scores is 0.264 mm. On the Spoon No 3548 (Fig 14A, 14B, 14E, 14F, 14I, 14J, 14K, 14M, 14N and 14O) (data in S2 Dataset), 143 scores and 741 pits, and on the Spoon No 6901 (Fig 15C, 15D, 15G, 15H, 15L and 15P) (data in S2 Dataset), 193 scores and 597 pits were detected. On the Spoon No 3549 (Fig 15A–15H) (data in S2 Dataset), 102 scores and 454 pits were detected.

*Third*, we analysed 357 marks on ten Neolithic tools (other than spoons) from the site of Vinča-Belo Brdo, i.e. 315 pits and 42 scores (data in S3 Dataset). The mean length, breadth, and L/B ratio values for pits are 2.01, 1.18, and 1.92 mm, respectively. The mean breadth of scores is 0.745 mm. The analysis of experimentally produced primary teeth marks, the marks on Neolithic spoons and on other Neolithic tools enable a comparison of the morphometrical characteristics of these three groups of marks.

### Taphonomical considerations

Many different agents, both recent and ancient, could have created marks on the Neolithic spoons and tools. The possibility that the marks are recent was, with a high level of certainty, ruled out in our study by applying the following two criteria: (i) the marks are of the same colour as the surrounding bone, and (ii) the edges of incisions are smooth (in contrast to jagged edges produced by recent damage) [56]. The ancient marks could have been created by numerous factors, such as intentional human modification of the bone in the course of tool manufacture or usage, the processes of weathering, trampling, biogenic damage, like plant root etching and animal teeth marks, and through biochemical processes by fungi and bacteria [48].

In order to distinguish between the marks produced by primary teeth and other marks, we conducted an experimental study in which 3151 primary teeth marks were analysed, providing explicit metrical and morphological characteristics of this specific type of mark. As we have shown, the experimentally produced primary teeth marks are significantly different in their dimensions from the marks on other bone tools and very similar to the marks on spoons. As it is reasonable to assume that other classes of tools were not used in any activity that would involve human teeth, the marks found on these tools represent traces of use, or any type of variable taphonomic traces. If the marks on spoons were generated by a similar agent (e.g. taphonomic), we would expect to have a metrical overlap between the marks on spoons and marks on other tools. As this is not the case, this gives us a basis on which to argue that different agents were involved in the creation of marks on the two classes of bone tools (spoons vs. other tools). Moreover, the density of marks is much higher on spoons than on other bone tools, and the metrical dimensions of spoon marks are within the range of experimentally produced marks. This does not mean that each of the 2230 marks detected on spoons were produced by primary teeth, but we provide clear evidence that, from the statistical point of view and in terms of metrical and morphological characteristics, the marks on spoons correspond to the marks made by primary teeth (unlike marks from other bone tools).

In our experimental study, primary teeth produced two types of marks–pits and scores − and those two types of marks were analysed on prehistoric spoons. These two types of marks could have also been created by other factors: score mark by intentional human modification of the bone with (sharp) tools, or by trampling and weathering, and the pit marks by animal teeth or through biochemical processes involving fungi and bacteria [48].

The scores produced by teeth have similar characteristics as the cut marks produced by stone tools and described by Shipman and Rose [57]: linear to slightly curved striations, parallel internal striations and a V-shaped cross-section. But there are two important distinctions between the scores produced by teeth and by stone tools: 1) the ‘shoulder effect’ visible in cut marks made by tools as a mark parallel to the cut mark made by irregularities of the edge of a stone tool is not present near the scores on spoons; 2) a V-shaped cross-section of the marks is only sporadically present in the case of spoons and was probably produced by teeth with a sharp edge such as canines, or by sharp molar cusps; however, in many scores on the spoons, a V-shaped cross-section is not present, probably when the score is produced by flat teeth such as incisors. Additionally, it is hard to understand why bone spoons would be damaged by stone tools in so many places and in very different parts of the spoons.

The pit marks of geometrical shapes found in our experimental study and on the spoons can also be created by animal teeth and fungi. Animal activity can be excluded because animals normally chew bones to get to the marrow, which is not present in spoons. The growth of fungi and bacteria on bones can create geometrical shapes such as elliptical and round, but this process is connected with discoloration, which enables the distinction between pits created by fungi from pits caused by other agents. Probable traces of fungal activity were indeed detected on spoons, in the form of clear discoloration of the pits; these pits were thus not measured.

**Fig 16:**
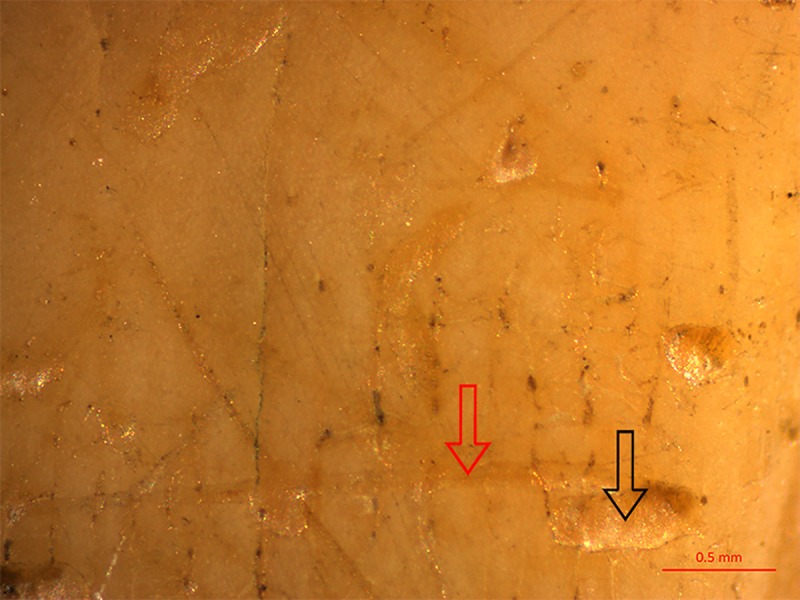
A pit and score on Spoon No. 03–3548. Probable activity of a single tooth, which, as a continual movement, created a pit by biting (black arrow) and a score by dragging the spoon (red arrow).

Our hypothesis that the majority of marks detected on spoons represent primary teeth marks is additionally supported by the fact that, in many cases, both types of marks, pits and scores were present simultaneously, probably representing traces of a single tooth, combining the activity of dragging the spoon over the teeth (creating score marks) and biting into it or gnawing on it (creating pit marks) (Fig 16).

### The absolute chronology of ten Early Neolithic bone spoons

**Fig 17:**
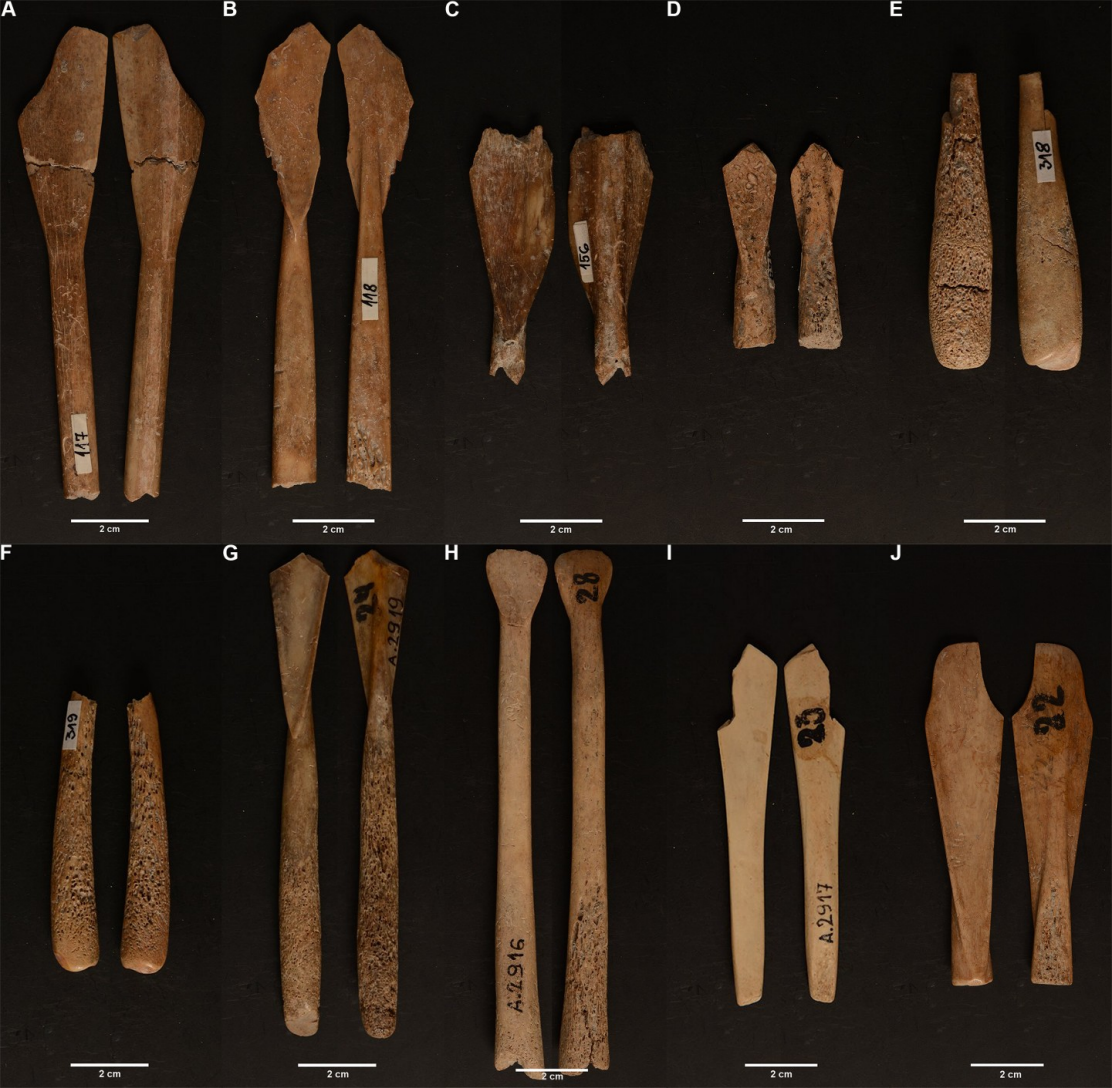
Spoons with C14 date. Spoons from Donja Branjevina (A) the Spoon 117, front and back, (B) spoon 118 (front and back), (C) spoon 156 front and back, (D) spoon 159 front and back, (E) spoon 318, front and back, (F) spoon 319, front and back. Spoons from Grad Starčevo (G) spoon 24 front and back, (I) spoon 28 front and back, (J) spoon 22 front and back.

Within the ERC BIRTH project’s major dating program, 10 Neolithic bone spoons were AMS dated, 6 of them from the site Donja Branjevina (DBR 118, DBR 319, DBR 117, DBR 318, DBR 156, and DBR 159) and 4 from the Starčevo-Grad site (Inv. no. 22 or 42, inv. no. 23/2917, inv. no. 24/2919, and inv. no. 28/2916) (data in S4 Dataset; Fig 17)

All the dates were calibrated in the OxCal 4.3 radiocarbon calibration program [58, 59]. The dates on spoons from Donja Branjevina indicate their use from around 5800 cal BC (5865 cal BC being the median value of 95.4% confidence interval for the oldest date) until ~5700 cal BC (5718 cal BC being the median value of 95.4% confidence interval of the youngest date). The earliest date from the Early Neolithic settlement at Donja Branjevina is around 6000 cal BC (6016 cal BC being the median value of 95.4% confidence interval for the oldest date). The youngest date on the spoon is also the youngest date currently available from the entire site, which could indicate a continuous use of this type of bone tools at Donja Branjevina, probably starting sometime after the establishing of life at the settlement, until its end.

The dates on bone spoons from Starčevo-Grad site indicate their use from ~5700 cal BC (5742 cal BC being the median value of 95.4% confidence interval for the oldest date) until ~5580 cal BC (5587 cal BC being the median value of 95.4% confidence interval of the youngest date). Starčevo-Grad is among the most dated Early Neolithic sites in the Central Balkans– 35 dates are currently available, suggesting the occupation from around 5900 cal BC until around 5500 cal BC. Even though the sample of dated spoons is small, it could be noted that their chronological span covers at least half of the period of the settlement duration.

## Discussion

The results clearly suggest that the marks found on spoons meet the two main criteria to be interpreted as tooth marks made by children: they conform in size and shape to the experimentally derived primary teeth marks and are different in density/frequency and size from the marks found on other classes of Neolithic bone tools. In addition to these main criteria, the distribution of the marks on spoons also meets the expectation that there will be marks on both sides of spoons in roughly corresponding areas. The discovery of children teeth marks on Neolithic spoons from Grad-Starčevo revealed that the main, if not the only function of these artefacts was baby-feeding. Numerous marks on a single spoon, on the recipient but occasionally also on the handle, are probably not a consequence solely of eating but represent a whole range of usual mouthing actions during the weaning period, such as biting, nibbling and gnawing. Although only three spoons were analysed within this study, it is reasonable to assume that many other spoons had the same function, given their uniform pattern of numerous traces of use described in the archaeological literature [26, 28–31]. The abundance of spoons throughout the Neolithic world indicates that this new type of artefact for baby-feeding was a response to the introduction of new types of weaning food. This discovery is important because it provides archaeological evidence of the existence of new infant feeding practices starting from the Neolithic, which adds to our understanding of the distribution and chronology of the appearance of new weaning foods in human history. In addition, this discovery suggests that the changes in feeding probably had a significant influence on prehistoric motherhood, which we hope will renew the interest and open new discussion on the role of breastfeeding and weaning in the advent of the Neolithic.

This discovery is important because it provides archaeological evidence of the existence of new infant feeding practices starting from the Neolithic, which adds to our understanding of the distribution and chronology of the appearance of new weaning foods in human history. In addition, this discovery suggests that the changes in feeding probably had a significant influence on prehistoric motherhood, which we hope will renew the interest and open new discussion on the role of breastfeeding and weaning in the advent of the Neolithic.

### The spoon chronology: The possibility for the reconstruction of the timing of infant feeding (re)evolution

Although spoons appeared before the Neolithic, there are only rare and isolated occurrences. Nandris mentioned a single Paleolithic bone object interpreted as a spoon [26]. The earliest secure find of a pre-Neolithic spoon was documented at a Geometric Kebaran site of ‘Uyun al-Hammam (context dated to ~16.5ky ago) [60]. Bone spoons were also present in the Natufian (~14.5–11.5 ky ago) [61, 62] and in Mesolithic Europe in the material culture of circum-Baltic hunter-gatherers [63]. However, the ubiquity and quantity of spoons in bone tool assemblages significantly increases in the Neolithic period, especially in the Early Neolithic of Anatolia and the Balkans [64, 65], and they are primarily a Neolithic phenomenon. Although bone tool industry has a long tradition, the appearance of a ‘spoon industry’ for infant feeding in the Neolithic can be seen as a reflection of the need to feed infants with a new type of weaning food. Thus, the future dating of spoons from Anatolia to Europe could establish the absolute chronology of the appearance and spread of this important invention in the history of infant feeding. In order to initiate the establishment of absolute chronology of the appearance of new types of tools and consequently new weaning food in the Balkans, we dated 10 spoons from two Early Neolithic sites in Serbia. So far, the dates point out that weaning food was not consumed with spoons or was not present in this region before 5800 cal BC. At this time, spoons appeared for the first time at the site of Donja Branjevina, and c. 5700 cal BC at the site of Grad-Stračevo, in both cases c. 200 years after the settlements were first established. In our sample, the youngest spoon, originating from the site of Grad-Stračevo, is dated to 5580 cal BC. However, this date corresponds to the end of use of the site, so it does not necessarily signify that it was the end of spoon use in the Neolithic Balkans. The same is true in the case of the youngest spoon from Donja Branjevina, dated to c. 5700 cal BC, corresponding to the end of the use of the site. According to dates from spoons available at the moment, new weaning food appeared in the Balkans c. 5800 cal BC. By considering the foodstuffs available in the Central Balkans during the Neolithic, we can hypothesize what were the main ingredients of weaning food.

### The main ingredients and the value of new weaning food?

Apart from their small dimensions, the bowls of Neolithic spoons are usually shallow, indicating a semi-liquid type of porridge. But what were the main ingredients of such porridges? Considering new foodstuffs that appeared in the Neolithic, milk and ground cereals would have been a good choice for gruel–they are easy to prepare and would have probably been available year-round. Animal milk would have been available in South-East Europe from between 9000 and 8000 years ago, with the appearance of the first economically important domestic animals [65]. The regional distribution of these herd animals varied but cattle was prevalent in the Central Balkans, as manifested by faunal assemblages from the Early Neolithic sites of Grad-Starčevo and Divostin [39, 65]. Pottery residue analysis suggests that milk was consumed as early as 8000 years ago, and the practice seems primarily related to the regions where cattle herding played a more important role [66]. Cattle could have been particularly valued for the amount of meat and milk they provided, and their physical strength. The choice of making bone spoons exclusively from cattle metapodials also leads to the question of whether this practice was related to the animal’s significant economic and symbolic role. Domesticated cereals became available between 9000 and 8500 years ago, when they first appeared in South-East Europe [67] and, several centuries later, in the central Balkans [e.g. 68]. In this region, the earliest remains of domestic cereals are of einkorn, emmer and barley [69, 70]. Finds of querns and grinding slabs at some of the Early Neolithic sites in the area [71] indicate the preparation of (cereal) grain foods, although very small quantities of crop remains (and plants in general) were recovered at the majority of these sites. Since milk and cereals were already present when the spoons appeared, it is plausible that those were the main ingredients of the new baby food. The appearance of new nutritional choices in child feeding in the Neolithic Balkans is also indicated by isotopic studies. Stable isotope ratios (δ^15^N, δ^13^C, δ^34^S) analysed on 25 Mesolithic and Neolithic children from the micro region of the Danube Gorges, showed different weaning food choices and also that Neolithic children were breastfed for a shorter time than Mesolithic ones. In addition, this study has shown that the period of exclusive suckling lasted on the average up to 1–2 years (the age at the introduction of solid food) in the Mesolithic period, and only around 6 months, in some cases even less than 6 months for the Neolithic children [72, 24]. Shorter breastfeeding, as indicated so far by the isotopic study for this region, was probably possible due to the introduction of new types of food for prehistoric babies. Moreover, a mass production of such foodstuffs can be assumed, given our new results concerning the function of the widely distributed Neolithic bone spoons. The appearance of alternative food choices could have had a profound impact on the whole process of motherhood and child care in the Neolithic.

The skill and time invested in the production of a single spoon could perhaps be indicative of the value placed on new weaing food in the Neolithic. As shown by experimental work conducted by I. Sidéra, who produced a replica of an Early Neolithic bone spoon, their manufacture demanded high skill and knowledge. Moreover, the operational formulas used for making spoons were sophisticated and technically complex [73]. She estimated that the production of a single spoon required approximately 25 hours of work, as well as the supervision or commented demonstration by experienced craftsmen [73]. These results could perhaps be interpreted as an additional indicator of the value placed on foodstuffs served by spoons, and/or on their consumers in the Neolithic. As previously noted, there has been a long history of negligence of women and children in archaeological research, and consequently the phenomena which integrates them both, such as prehistoric motherhood, have only recently started to be explored. A greater emphasis on motherhood in prehistory will hopefully lead to a better recognition of artefacts or architectural features of dwellings and settlements which were related to birthing and child care. As we showed with the discovery of the function of Neolithic bone spoons, the negligence of phenomena related to motherhood, as part of everyday life of people in prehistory, led to the failure to recognize these artefacts as part of the new Neolithic weaning equipment, in spite of their resemblance to spoons used to feed children to this day. In the light of the high density and wide distribution of spoons, our results could suggest that new kinds of gruel were also an important part of the ‘Neolithic package’ or rather the ‘Neolithic motherhood package’.

### The discovery of spoons for feeding babies and the ‘weaning food availability’ hypothesis’

The currently available evidence of new weaning food in the Central Balkans, at the latest around 5800 BC according to the absolute chronology of spoons, as well as the shorter breastfeeding of Neolithic mothers demonstrated by the isotopic study, support the previously rejected ‘weaning food availability hypothesis’. Ultimately, this new evidence could renew and stimulate the discussion on the influence of new infant food choices on the duration of breastfeeding. Moreover, it could also trigger the discussion on the possibilities of new kinds of organisation of baby care, given that new, ‘easy to prepare’ types of gruel probably allowed other persons to be involved in baby weaning, to a much greater extent than before the Neolithic.

The correlation between agricultural food and weaning was originally discussed by Binford, who suggested that weaning of children starts at an earlier age in agricultural and pastoral societies because of the greater availability of starchy products, which are more suitable in early weaning in comparison to the more fibrous wild foodstuffs available to hunter-gatherers [74]. This hypothesis was further developed by suggesting that one of the causes of the fertility increase may have been connected to the reduction in breastfeeding as a consequence of the appearance of new agricultural foods, suitable for feeding infants [15, 17]. However, this assumption was abandoned, mainly due to the study of Sellen and Smay [18], who analysed ethnographic data from agricultural and foraging populations and found that both use a wide variety of weaning foods, and that foragers even introduce liquids and solids in infant diet earlier than agricultural populations. On the other hand, they did find that the duration of breastfeeding was relatively short in agricultural populations compared to the foragers, who were breastfeeding for a longer time. Moreover, these authors did not exclude the possibility that the fertility increase was caused by the reduction of breastfeeding. But, they rejected the possibility that the duration of breastfeeding was influenced by the availability of weaning food in agricultural populations, and that the causes for its reduction should be explored in other aspects of Neolithic life, for example the changes in female working patterns as the duration of breastfeeding is under the direct influence of tasks and activities performed by mothers [18, 75]. Although this ethnographic data is valuable without any doubt, we argue that there is still not enough substantial evidence to reject the ‘weaning food availability’ hypothesis, for at least three reasons. First of all, even with the growing number of bioarchaeological studies on weaning, the studies including a larger sample of both Mesolithic and Early Neolithic children are rare and there is still no sufficient data on the duration of breastfeeding or the introduction of weaning foods based on direct skeletal evidence. Consequently, much more evidence is needed in order to make conclusions about possible changes in the duration of breastfeeding and the role of new weaning food during the Neolithic transition. Second, the ‘weaning food availability’ hypothesis was discussed in the context of female breastfeeding, but rarely explored in the light of possible changes involving new types of gruel, which allowed persons other than the mother to be included in baby feeding. Further research into the mechanisms of weaning in the Neolithic could explore the participation of other persons in childcare and their potential role in the increase in child survival rate. Third, the entire ‘weaning food availability’ discussion omits the possible role of new Neolithic foodstuffs, especially the role of animal milk as a substitute for breast milk during the stage of infant life which would have been the period of exclusive suckling during the Mesolithic. Thus, for the first time in human history, the use of animal milk enabled artificial feeding of newborn babies whose mothers died or had lactation problems. In addition, animal milk also provided opportunities for mothers without lactation problems to choose not to breastfeed or not to breastfeed as often. On the one hand, it is not surprising that the ‘weaning food availability’ discussion ignored the period of exclusive suckling, because the weaning process began with the first introduction of foods other than breast milk and ended with the cessation of breastfeeding. But the focus on weaning food only as supplementary food to breast milk and not as a potential substitution for breast milk, ignored the role of animal milk as a possible option for feeding small babies before they can digest food other than mother’s milk. Although one can agree that the opportunities for providing a variety of weaning food were similar in farming and forager communities, Neolithic mothers were the first mothers in human history who had the opportunity to provide their babies with a substitute for breast milk.

### To feed or to breastfeed–a new choice for Neolithic mothers

For the first time in human evolution, the opportunity to feed babies with animal milk, especially in cases of death of the mother during or shortly after childbirth, probably had an important impact on child survival. Although the maternal mortality rate in prehistory is not known, it was probably very high. Given that the maternal mortality rate is still high even in the XXI century, for example in 2015 around 303 000 women died during and following pregnancy and childbirth [76], we can assume that many prehistoric babies lost their mothers due to birthing difficulties. Unfortunately, the consequences of the death of a mother during the early stages in the life of a baby are catastrophic, and only a small percent of children survive if their mothers die during childbirth (for example, only 1.6% of Swedish children survived in cases of maternal death in the 19th century) [77]. Thus, we can assume that the survival rate of Neolithic babies whose mothers died giving birth increased due to the availability of animal milk to substitute mother’s milk. Moreover, animal milk afforded not only a better chance for survival of babies who did not have the opportunity to consume breast milk, but also as an opportunity for Neolithic mothers without lactating difficulties to choose not to breastfeed or to breastfeed infrequently. Whether and to what extent they used this opportunity will hopefully be better understood with future isotopic studies, as well as with future archaeological investigations because feeding small babies animal milk potentially left certain traces on artefacts. In the meantime, although it is not entirely adequate to compare the Neolithic to the modern age, some important indications of willingness of mothers to accept novelties in newborn baby feeding practices and to use substitutes can be gained by observing the advent of baby formulas at the beginning of the XX century. When artificial baby milk started to become widely advertised in the USA by the early 1900s, marketing campaigns promised mothers ‘more freedom than they had ever known’ [78, 79]. At the time, breastfeeding rates were 95% with weaning occurring at 2–3 years of age, but by the 1960s breastfeeding rates significantly dropped to less than 25% [79, 80]. Certainly, probably many other factors contributed to the decrease of ‘breastfeeding moms’, but obviously the simple possibility of using artificial milk led to a large number of women choosing not to breastfeed. Although it is highly speculative, it can be assumed that the possibility of using animal milk instead of one’s own, at least on some occasions, might have been appealing to Neolithic mothers as well. The implications could have been important even for mothers who choose to use animal milk instead of their own only occasionally. If that was the case, the consequences can be assumed from what is known on female physiology concerning the connection between child suckling and lactation. Namely, when the baby suckles, it sends a signal to the pituitary gland which activates the secretion of prolactin and oxytocin, meaning that the amount of released hormones will depend on the frequency, intensity and duration of suckling [81, 82]. As the hormone prolactin regulates female lactation, the connection between suckling and hormone levels means that the reduction of breastfeeding will drop the level of hormones which have direct influence on further lactation, and will cause insufficient milk production [83]. Consequently, if Neolithic mothers started to use animal milk as a substitute, even occasionally, during the period when babies were exclusively breastfed in pre-Neolithic times, they could have experienced lactation problems and a reduction of lactation. But, at the same time, decreasing levels of prolactin and oxytocin could have influenced a fertility increase, given that those hormones also regulate ovulation and that is why breastfeeding indirectly suppresses ovulation. Because of that, the potential use of animal milk as a substitute for breast milk could have had a negative effect on lactation, but a positive effect on fertility increase during the Neolithic. Since Neolithic communities had access to dairy products, it seems unlikely that there would have been taboos related to the usage of animal milk in infant diet instead of mother’s milk. It is actually possible that infants were the first consumers of animal milk [84], as genetic evidence suggests that adults were not capable of digesting animal milk at the time, because the mutation which would enable it was very rare among Neolithic people [85].

Rather than ignoring negative health consequences, especially in cases of the usage of raw milk in the diet of newborns, which are discussed elsewhere in detail [84], our intention is to underline the importance of further research in this direction. To begin with, the ‘weaning food availability’ hypothesis should be expanded to consider the ‘breast milk substitute availability’ by investigating the role of animal milk as an alternative for breast milk with the advent of the Neolithic.

### The changes in prehistoric motherhood with the Neolithic

When first formulated, the ‘weaning food availability’ hypothesis tried to explain the connections between the availability of new types of weaning food which appeared in the Neolithic and the duration of breastfeeding. But the role of new food for prehistoric babies should be considered not only in connection to mothers and the duration of breastfeeding, but also in a wider context of prehistoric child care. Since being a mother does not imply that women are alone in child care, as humans, as well as many other species, have a cooperative breeding system, this means that other, non-parental individuals also actively participate in caring for the offspring [86]. As the saying goes, it takes a village to raise a child—but who keeps the children alive, to quote the question by R. Sear who analysed the effects of kin in child survival. In her study of both historical and contemporary populations across a wide geographical range, she found that various maternal and paternal relatives have important but distinct roles in providing help to the mothers. The most beneficial for improving child survival chances are maternal grandmothers and to some extent sibling helpers [77]. It is shown that maternal grandmothers seem to play a key part during weaning by giving advice and practical support, which provides a mechanism for increasing child survival rates [77, 87]. The whole mother support system implies that in order to understand the fertility increase in the Neolithic, we need to investigate the contributions of different Neolithic innovations to the changes in cooperative breeding. For this reason, we cannot discuss the ‘weaning food availability’ hypothesis only from the perspective of the reduction of lactation in mothers, but also in a wider context of potential changes to the cooperative breeding system brought by new baby food. We argue that new kinds of baby food, such as animal milk and different types of gruel made from cereal grains could have had profound effects on the whole system of prehistoric child care. We suggest that new kinds of baby food were an important part of the ‘Neolithic motherhood package’ which changed the previous system of cooperative breeding, for several reasons. First, according to the density and wide distribution of bone spoons which were most likely used to serve new kinds of gruel, it appears that this innovation was widely accepted by Neolithic communities, which would not be the case if they were not convinced of its usefulness. Second, this new type of baby food is a great innovation compared to all weaning choices before the Neolithic. Although many studies have shown that foraging populations may use a variety of weaning foods, when it comes to the availability of baby food it is difficult to find something which can compare to new Neolithic gruels. If we assume that their main ingredients were animal milk and cereals, it is obvious that milk could have been available at all times but with Neolithic storage practices the same could have been true with cereals. With these two ingredients, one needed only a grinding slab, even a small one, to prepare a baby meal. Of course, new kinds of gruel could have also been served by wooden spoons and probably in many cases were, and the bone spoons were probably not necessary but were nonetheless skilfully made and a fine utensil to fed babies with. In addition, the appearance of a completely new type of bone tool is indicative of new baby foods and a need for a new kind of tool to serve it. Finally, new baby food provided everyone (including children) with opportunities to prepare baby meals easily, during all seasons including winter, during the day or night, without foraging. For these reasons we assume that new baby food was not only beneficial to the mothers, influencing or not their lactation, but was important for all persons who were engaged as helpers. As cooperative breeding in humans always existed but role of specific helpers is subjected to change, we suggest that the opportunity of each helper to contribute to baby care on a greater scale than ever before could have had profound effects on the survival of children with the advent of the Neolithic.

### Possible negative consequences

Although we suggest that new baby food was an important innovation in prehistoric motherhood, and probably one of the factors which caused the fertility increase, there are also some negative consequences of such food which also need to be investigated in the future. There are at least two possible consequences of new feeding practices: 1) the health risk of using raw animal milk in infant diet, 2) the risk of bacterial infections from using unclean feeding utensils. But, similarly to the invention of baby formula in the beginning of the XX century which was quickly accepted by many mothers, it probably also took some time for the Neolithic communities to comprehend the negative consequences of a new diet for their babies.

The most negative effect of milk in infant diet would manifest in cases of use of raw ruminant milk, which could cause infectious illnesses and a greater risk of iron-deficiency anaemia, but the magnitude of risk would depend on infant age and the amount of animal milk in their diet [84]. Another important health issue was probably related to the hygiene of the ‘weaning equipment’, because utensils used for storage, preparing and serving food could have developed bacteria detrimental to infant health. As it is known from the early XIX century, the use of unclean feeding utensils and the lack of proper milk storage and sterilization were responsible for the deaths of one third of all artificially fed infants during their first year of life [88].

The consequences of the use of raw milk (fermented milk is good for infants) and unclean feeding utensils, from infectious diseases to iron deficiency, could probably be one of the important causes of the health decline in Neolithic populations [89–92], but it did not stop humans from using animal milk and cereals in children diet to this day.

Finally, we can conclude that the Neolithic brought about important changes in infant feeding practices, as demonstrated by the high density and wide distribution of bone spoons which served as baby feeding utensils. That new feeding choices brought by Neolithisation remain important for the prehistoric infants is recently shown by the discovery of the milk products from ruminants found in the clay ‘baby bottles’ from the Bronze Age [93]. We suggest that this innovation of ‘easy to obtain and prepare’ baby meal made profound changes in prehistoric motherhood, allowing other persons to participate as helpers in child care more intensively. These changes probably brought important improvements in prehistoric motherhood, with potential positive effects on the fertility increase in the Neolithic, but much more studies are needed in order to understand both biology and culture behind this turning point in human history. From our perspective, the most important tasks for future studies on the causes of the fertility increase include the investigation of (bio)archaeological evidence of the organization of child care, breastfeeding and weaning practices in order to understand how the Neolithic changed prehistoric motherhood. Although it is true that it takes a village to raise a child, it is the task of archaeology and bioarchaeology to provide a better understanding into the ways Neolithic villagers were organized which enabled them to raise more children than any populations before.

## Supporting information

S1 Movie A 3D model of a Neolithic spoon. (MP4)

S1 Fig The excavation and dating of Grad-Starčevo. (A) Excavated tranches at Grad-Starčevo site; (B) the distribution of 17 radiocarbon dates.
(TIF)

S1 Text The spoons from the Grad-Starčevo site and other analysed Neolithic tools and with sentence the beginning of the occupation can be dated to 5770–5565 cal BC (95% probability), whereas the Late Neolithic sequence lasted until 4570–4460 cal BC (95% probability) [94]. (DOCX)

S1 Dataset The measurements of experimentally produced marks of primary teeth. (XLSX)

S2 Dataset The measurements of marks on spoons. (XLSX)

S3 Dataset The measurements of marks on tools. (XLSX)

S4 Dataset Double-sidedness of the marks on spoons. (XLSX)

S5 Dataset Dated bone spoons. (XLSX)
