# Supplementary figures and images for: Bone spoons for prehistoric babies: Detection of human teeth marks on the Neolithic artefacts from the site Grad-Starčevo (Serbia)
Source: PLoS One. 2019 Dec 19;14(12):e0225713. doi: 10.1371/journal.pone.0225713 (PMC6922321; doi:10.1371/journal.pone.0225713)

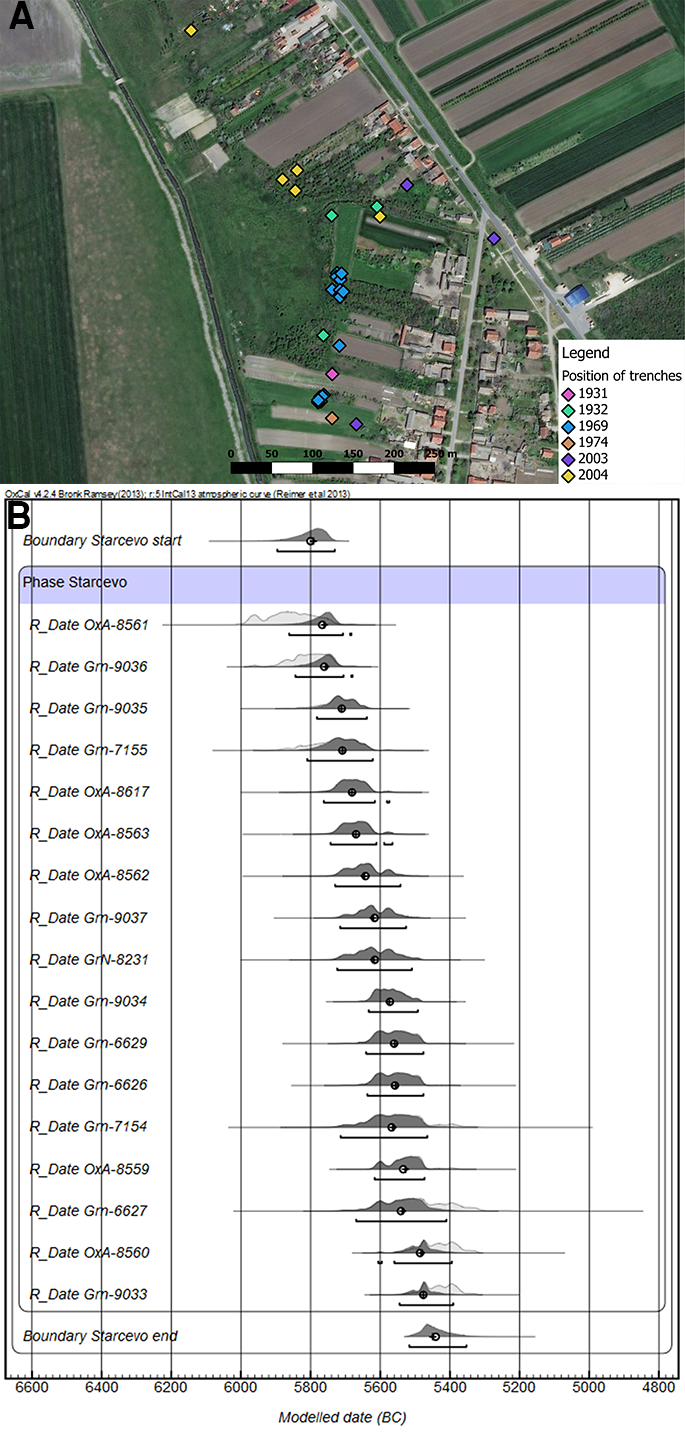

Supplement: S1 Fig — (A) Excavated tranches at Grad-Starčevo site; (B) the distribution of 17 radiocarbon dates. (TIF) [file pone.0225713.s002.tif]
