# Supplementary material for: Bone spoons for prehistoric babies: Detection of human teeth marks on the Neolithic artefacts from the site Grad-Starčevo (Serbia)
Source: PLoS One. 2019 Dec 19;14(12):e0225713. doi: 10.1371/journal.pone.0225713 (PMC6922321; doi:10.1371/journal.pone.0225713)
Supplement: S1 Text — (DOCX) [file pone.0225713.s003.docx]

## **Spoons from the Grad-Starčevo site**

For the spoons and tools analysed within this study, the greatest length (GL) and greatest breadth (GB) were measured.

Spoon No. 03 - 3548 – (*preserved length=113.5 mm, GL bowl=53.6 mm, GB bowl=17.8 mm, GL handle=56.3 mm, GB handle=10.8 mm*) – Bone spoon with a slightly concave elongated asymmetrical bowl, V-shaped junction of the bowl and handle, and a cylindrical handle.  The bowl is of asymmetrical shape and its edge had been broken.

Spoon No. 03-3549 – (*preserved length=88.4 mm, preserved length of handle=62.4*) – Bone spoon with bowl broken near the smooth junction and with a cylindrical handle (also broken).

Spoon No. 03-6901 – (*GL=143.3 mm, GL bowl=51.2 mm, GB bowl=19.6 mm, GL handle=94.2 mm, GB handle=12.3 mm*) – Complete bone spoon; the bowl is slightly concave, elongated and asymmetrical, with one broken corner. The handle is curved and cylindrical in cross-section, in particular towards the base.

## **Other analysed Neolithic tools**

Neolithic bone tools other than spoons analysed in this study originate from the Vinča-Belo Brdo site. Vinča is a tell-type settlement and one of the largest in the Central Balkans. The wealth of excavated material and the long duration of the site made it a yardstick for the sequencing of the Neolithic in the region. The beginning of the occupation can be dated to 5770 – 5565 cal BC (95% probability), whereas the Late Neolithic sequence lasted until 4570–4460 cal BC (95% probability) [41]. The bone tool assemblage from Vinča-Belo Brdo analysed within this study site consists of pointed tools – awls, a burnisher and two fish hooks, one of which is a lure hook.

E(letronic) D(istance) M(easurment) 24 (2008) – (*GL=62.4 mm, GB=6.3 mm) –* Awl made of the diaphysis of a long bone of a medium-sized ungulate. The bone was longitudinally split, while the pointed shape was obtained by cutting and polishing. The basal part was broken off, probably to be inserted into a grip.

EDM 28 (2009) – (*GL=80 mm, GB=7.1 mm) –* Awl made of the diaphysis of a long bone of a medium-sized ungulate. The bone was longitudinally split, whereas both its flat base and pointed shape were obtained by cutting and polishing.

EDM 387 (2005) – (*GL=79.5 mm, GB=7.7 mm) –* Awl made of the diaphysis of a long bone of a medium-sized ungulate. The bone was longitudinally split, whereas both its flat base and pointed shape were obtained by cutting and polishing.

EDM 467 (2004) – (*GL=75.9 mm, GB=10.5 mm)* – Awl made of a rib of a large-sized mammal. The rib was longitudinally split, whereas both its flat base and pointed shape were obtained by cutting, burnishing and polishing.

EDM 238 (2009) – (*preserved length=95.9 mm, GB=11.3 mm) –* Awl made of a rib of a large-sized mammal. The rib had been longitudinally split, while its pointed shape was obtained by cutting and polishing. The basal end is broken.

EDM 64 (2004) – (*GL=30.2 mm, GB=8.2 mm) –* Awl made of the diaphysis of a long bone of a medium-sized ungulate. The bone was longitudinally split, while the pointed shape was obtained by cutting and polishing. The basal part was broken off, probably to be inserted into the grip, while its point was broken and reused.

EDM +386 (2005) – (*GL=51.7 mm, GB=12.6 mm) –* Awl made of the proximal part of a roe deer metatarsal bone. The bone was longitudinally split, while the pointed shape was obtained by cutting and polishing. The basal part was made of the proximal epiphysis. The working tip is not pointed – it had probably been broken, but the use of the tool continued.

EDM 33 (2008) – (*GL=117.6 mm, GB=13.1 mm)* – Burnisher made of the diaphysis of a long bone of a large-sized mammal. The bone was longitudinally split, while the shape of both ends was obtained by cutting and burnishing. The tool is elongated, one of its ends is oval, while the other is pointed. The entire surface of the inner diaphysis was probably used.

EDM 260 (2009) – (*preserved length=43.8 mm, preserved breadth=5 mm) –* Fragmented fish hook made by cutting and fine polishing of a mammal long bone. The basal part, with a groove for attaching the fibre, is preserved, while the point is missing.

EDM +158 (2006) – (*GL=37.4* *mm, GB=13 mm)* – Lure hook made by the longitudinally splitting and cutting of a rib of a large mammal. The notch was obtained by drilling and the basal part was not worked.
